# Immunotherapeutic Strategies as Potential Treatment Options for Cutaneous Leishmaniasis

**DOI:** 10.3390/vaccines12101179

**Published:** 2024-10-17

**Authors:** Andrea Lafleur, Stephane Daffis, Charles Mowbray, Byron Arana

**Affiliations:** 1 Doctoral Training Centre, University of Oxford, Oxford OX1 3NP, UK; 2 Drugs for Neglected Diseases initiative (DNDi), 1202 Geneva, Switzerland; sdaffis@dndi.org (S.D.);

**Keywords:** cutaneous leishmaniasis, immunotherapy, TLR agonism, therapeutic vaccination

## Abstract

Cutaneous leishmaniasis (CL), caused by protozoan parasites of the *Leishmania* genus, is prevalent in tropical and subtropical regions, with important morbidity, particularly in low- to middle-income countries. Current systemic treatments, including pentavalent antimonials and miltefosine, are associated with significant toxicity, reduced efficacy, and are frequently ineffective in cases of severe or chronic CL. Immunotherapies leverage the immune system to combat microbial infection and offer a promising adjunct or alternative approach to the current standard of care for CL. However, the heterogeneous clinical presentation of CL, which is dependent on parasite species and host immunity, may require informed clinical intervention with immunotherapies. This review explores the clinical and immunological characteristics of CL, emphasising the current landscape of immunotherapies in in vivo models and clinical studies. Such immune-based interventions aim to modulate immune responses against *Leishmania*, with additive therapeutic effects enabling the efficacy of lower drug doses and decreasing the associated toxicity. Understanding the mechanisms that underlie immunotherapy for CL provides critical insights into developing safer and more effective treatments for this neglected tropical disease. Identifying suitable therapeutic candidates and establishing their safety and efficacy are essential steps in this process. However, the feasibility and utility of these treatments in resource-limited settings must also be considered, taking into account factors such as cost of production, temperature stability, and overall patient access.

## 1. Introduction

Leishmaniasis is a vector-borne disease caused by protozoan parasites of the *Leishmania* genus and is characterised by a large spectrum of clinical manifestations, including visceral leishmaniasis (VL) and cutaneous leishmaniasis (CL) [1,2,3]. CL is endemic to the tropics and subtropics, with approximately 600,000–1 million new cases reported annually—over 95% of which occur in South and Central America, the Mediterranean basin, and Western and Central Asia [4]. Though CL-associated mortality is low, an estimated 770,000 disability-adjusted life years (DALYs) are attributable to the disease [5]. Classified as a neglected tropical disease (NTD) by the World Health Organization (WHO) since 2007, CL poses a threat to an additional 350 million–1 billion individuals residing in endemic regions, and its incidence is widely considered to be underreported [4,6,7]. Alarmingly, as climate change enables the northward migration of the parasite’s vector—the phlebotomine sandfly—endemic regions are expected to expand in coming years [8].

The Pan American Health Organisation (PAHO) and the WHO recommend various treatments for CL [9]. First-line therapeutic interventions are determined based on clinical characteristics, including the number, size, and localisation of lesions, as well as the causative species of *Leishmania*. In the Americas, particularly Central and South America, PAHO recommends miltefosine as the primary systemic treatment option, whereas in Africa, Asia, Europe, and the Middle East, pentavalent antimonials are the standard treatment [9]. In both the Americas and elsewhere, topical CL treatments such as intralesional antimonials, thermotherapy, and cryotherapy are preferred for patients with small lesions (≤4 cm in diameter).

Parenteral administration of pentavalent antimonials is associated with severe adverse effects including pancytopenia, peripheral neuropathy, and nephrotoxicity [1]. Meglumine antimoniate (MA) and sodium stibogluconate (SSG) have similar efficacy and toxicity [1], although primary resistance has been reported in up to 15% of patients treated with pentavalent antimonials [10]. Oral miltefosine is associated with gastrointestinal side effects, including anorexia, nausea, vomiting, and diarrhoea, as well as more severe adverse effects, including skin allergies, elevated hepatic transaminase levels, and, less commonly, renal insufficiency and potential teratogenicity [11]. Moreover, clinical failure is reported in 10–20% of cases treated with miltefosine [1]. Second-line treatments, including paromomycin, pentamidine, and amphotericin B, also have significant nephrotoxicity, pancreatitis, cardiotoxicity, and teratogenicity [11]. Given the important global burden of CL, the development of effective and safe therapeutic interventions is crucial.

Immunotherapy is a treatment modality that modulates immune responses to resolve disease and has emerged as a promising therapeutic intervention for CL [12]. Herein, we describe the main clinical and immunological features of acute and complex CL and review studies exploring immunotherapies in vivo and in human studies. Specifically, we review and discuss the current state of immunotherapies, including recombinant cytokines, antagonists of cytokines, immune checkpoint inhibitors, TLR agonists, modulators of cellular receptors and signalling, enzyme inhibitors, anti-inflammatory and antioxidant agents, cellular therapy, and therapeutic vaccines.

## 2. Clinical and Immunological Features of Acute and Complex CL

CL presents with varying degrees of severity, resulting in a range of clinical outcomes. While many infections remain asymptomatic, CL is characterised by the appearance of a small erythema at the site of a sandfly bite, which ulcerates over subsequent weeks or months [1,2]. The most prevalent form of CL—localised cutaneous leishmaniasis (LCL)—presents acutely as a single or a limited number of ulcerated lesions, usually on the face, extremities, or other regions of the body that may be exposed to the environment, and can be caused by a wide variety of *Leishmania* species [1,13]. Though often self-limiting, LCL lesions can cause significant scarring and constitute a risk for secondary fungal and bacterial infections [13,14]. Furthermore, up to 10% of acute LCL cases progress to severe and chronic complex forms of CL, which present additional clinical challenges [4].

The outcome of CL is highly dependent on the host immune response, resulting in either parasitic clearance or acute/chronic infection [2]. *Leishmania* infection occurs when a sandfly harbouring the parasite takes a bloodmeal from a mammal (i.e., a human, rodent, or canid), egesting metacyclic promastigotes into the host dermis [2,15]. Phagocytic cells such as neutrophils, dendritic cells (DCs), and monocytes rapidly internalise the parasites, attempting to control infection by an initial burst of reactive oxygen species (ROS) and inflammatory cell recruitment [16,17]. Neutrophils can help clear infection using neutrophil extracellular traps (NETs) but may also contribute to macrophage infection through a “Trojan horse” mechanism [18].

The adaptive immune response is initiated after the presentation of *Leishmania* antigens, leading naïve T cells to differentiate into Th1 or Th2 effector CD4^+^ T cells, which determines macrophage polarisation and the course of CL [16]. Th1 cytokines (i.e., IL-12, IFN-γ, and TNF) activate M1 macrophages to produce leishmanicidal nitric oxide (NO) and ROS [19], enabling parasitic clearance, while Th2 cytokines (i.e., IL-4, IL-10, IL-13, and TGF-β) activate M2 macrophages, facilitating parasitic persistence [2,16,20]. A robust and coordinated Th1 immune response is recognised as one of the key effectors in mediating parasitic clearance and often leads to a clinical cure; however, excessive Th1 inflammation can cause tissue damage [18]. Conversely, while a Th2 response is associated with parasite persistence and immunotolerance, it also mitigates Th1-driven tissue damage and is essential for healing lesions [18].

Thus, both Th1 and Th2 cytokines are required for self-healing LCL [21], although dysfunctional or imbalanced Th1/Th2 immune responses can contribute to the immunopathology and development of complex CL. Persistent subclinical parasites, which enable continuous antigen presentation and maintenance of immune memory [18], may cause relapsing CL, with IL-10 and TGF-β implicated in infection recurrence [22]. Th17 responses, marked by IL-17 production, can aid in parasite control but also contribute to excessive inflammation and tissue damage, particularly through increased neutrophil recruitment, which can worsen clinical outcomes [23]. Furthermore, regulatory T cells (Tregs) in lesions suppress effector cytokine-producing cells, enabling the long-term persistence of parasites [24].

Leishmania recidivans (LR), a relapsing form of CL, involves repeated reappearance of cutaneous lesions near previous infection scars [13,25]. Local trauma or immunosuppression, such as with topical corticosteroids, can reactivate latent parasites [26,27], causing relapse multiple years after clinical cure [13,25]. During LR outbreaks, peripheral IFN-γ-producing CD4^+^ T cells decrease in favour of immunosuppressive CD8^+^ T cells, with cell populations restoring after treatment [26]. LR infiltrates contain Langhans giant cells (LGCs), a subset of granulomatous multinucleated macrophages [28,29,30], which may either promote parasitic survival through haemophagocytosis or reduce pathogen spread [31], as seen in tuberculosis [32,33,34]. Endemic to Iran, LR affects 5% of LCL cases and is largely resistant to first-line antileishmanials [25,35].

Anergic diffuse cutaneous leishmaniasis (ADCL) features slowly progressing plaques, nodules, or non-ulcerative lesions covering large body areas [36]. It results from an insufficient Th1 response and excessive Th2 response, with high IL-4, IL-5, and IL-10 levels contributing to alternative macrophage polarisation [37] and TGF-β release, inducing T cell anergy [1,38]. However, while the association of IL-10 and TGF-β in recurrence has been observed in murine models, their roles in human recurrence remain unclear. Certain *Leishmania* strains contribute to this feedback loop via lipophosphoglycan (LPG), which interacts with TLR4 on antigen-presenting cells (APCs) to promote Th2 differentiation and stimulates TLR9, reducing NO production by macrophages [36,39,40]. ADCL is also characterised by reduced NK cell populations and lower TLR1, TLR2, and TLR6 expression [41]. Treatments are largely ineffective, although antimonials can reduce lesion severity, and relapse is common [42,43,44].

Disseminated leishmaniasis (DL) is a metastatic form of CL, with an incidence of up to 3.9% in northeastern Brazil [45]. It is defined by 10 or more lesions across at least two body regions, spreading 2–6 weeks after an initial lesion [45]. DL can result in hundreds or thousands of lesions on the limbs and the face, with 53% involving the mucosa [45]. DL dissemination is linked to a lack of peripheral Th1 response, including reduced IFN-γ and TNF-α from peripheral blood mononuclear cells (PBMCs), allowing parasites to spread via the bloodstream [46]. Increased serum CXCL9 suggests that *Leishmania*-specific T cells migrate to lesion sites, reducing circulatory populations and systemic surveillance [47]. DL lesions exhibit local inflammation with macrophages, plasmacytes, and T cells [48], though lower B cell counts may explain their non-ulcerative nature [49]. Three-quarters of DL cases are unresponsive to pentavalent antimonial therapy, but prolonged treatment and liposomal amphotericin B can reduce lesion numbers and severity [45].

Although Th1 responses are the major effectors of parasite clearance, an excessive skewness towards these responses promotes an inflammatory phenotype that can be pathological [50,51]. This occurs in mucocutaneous leishmaniasis (MCL), another form of metastatic CL, where *L. (Viannia)* parasites migrate from a primary lesion to the nasopharyngeal mucosa, causing severe inflammation and permanent disfigurement [2,3]. MCL involves high cytotoxicity, excessive IFN-γ and TNF-α production, and low circulatory IL-10 levels [18]. During lesion ulceration, it is thought that CD8^+^ T cells, expressing high levels of cytotoxic effectors, including granzymes and the NKG2D-activating receptor, migrate to the site of infection and induce cell death [18,52,53]. MCL lesions also contain abnormally large populations of neutrophils, Th17 cells, plasma cells, and B cells, with high levels of IL-17-inducing cytokines, promoting inflammation, local apoptosis, and tissue damage [49,54]. Symptomatic parasitic metastasis is linked to high IL-17A and low IFN-γ [55]. Although mechanisms underlying parasitic metastasis in MCL remain unclear, increased expression of CXCL10 by *L. (Viannia) braziliensis*-infected PBMCs could contribute to parasitic dissemination to the mucosa similar to CXCL9 in DL [56]. MCL is treatment-refractory in up to 40% of cases, requiring secondary therapies and prolonged regimens, increasing the risk of cardiotoxicity, nephrotoxicity, and hepatotoxicity [57]. In regions of Brazil endemic to *L. (V) braziliensis*, 1–10% of LCL cases progress to MCL, with some LCL cases relapsing as MCL years later [58,59,60].

MCL and DL share regions of endemicity and are frequently caused by the same *Leishmania* species. While mechanisms underlying disease progression into MCL or DL generally remain elusive, several studies indicate that divergent parasite strains with genetic differences may induce stronger Th1 responses and mucosal involvement [61,62,63]. Infection of the MCL-causing *Leishmania* (*Viannia*) species by *Leishmania* RNA Virus 1 (LRV1), an endogenous double-stranded RNA totivirus, contributes to increased pathology and localised inflammation [55,64,65]. This occurs through the interaction of the viral dsRNA with mammalian endosomal TLR3 [66], triggering pro-inflammatory cascades and type I IFN production, which downregulates IFN-γ receptors on macrophages, reducing their antiparasitic response [67]. LRV1 also inhibits caspase-11 and IL-1β maturation, hindering inflammasome assembly [68,69]. Additionally, degradation of NLRP3 and ASC via autophagy further subverts the inflammasome, correlating with MCL severity [70].

Post-kala-azar dermal leishmaniasis (PKDL) presents as macular or papular lesions that can coalesce into plaques but rarely become ulcerative; they can be subdivided into monomorphic (single lesion type) and polymorphic (multiple lesion types) forms [71]. PKDL is a relapsing complication of VL, developing in 50–60% of Sudanese and 5–10% of Indian cases [72,73]. About 15–20% of PKDL cases result from subclinical VL, and the incidence is higher in HIV co-infected patients [74]. It is characterised by an unbalanced Th1 response typically occurring as a result of VL treatment, with decreased Treg populations and levels of TGF-β and IL-10, and excess pro-inflammatory cytokine production (IFN-γ, TNF-α, and IL-12) [75,76]. Activated *Leishmania*-reactive T cells infiltrate the skin, where low levels of *L. donovani* persist, releasing Th1 cytokines and causing lesions [77,78]. PKDL lesions have elevated Th17 responses [79,80], increased intralesional CD8^+^ T cells expressing programmed death-1 (PD-1) and exhaustion markers, and decreased CD4^+^ T cell populations [81]. Polymorphic lesions have higher levels of IFN-γ and TNF-α, NK cells, CD3^+^ T cells, and M2 macrophages with decreased levels of TLR2/4, ROS, and NO, compared to monomorphic PKDL [71,80,82]. PKDL treatment often fails with antimonials and first-line therapies, although spontaneous remission occurs in non-severe cases [83].

The immunopathogenesis of chronic and relapsing CL varies with the strength and timing of the immune response [84]. The same immune effector can be both pathogenic and crucial for disease resolution [84]. This variability complicates predicting infection outcomes and developing effective immunotherapeutic interventions.

## 3. Immunotherapies for Acute and Complex CL

**Figure 1:**
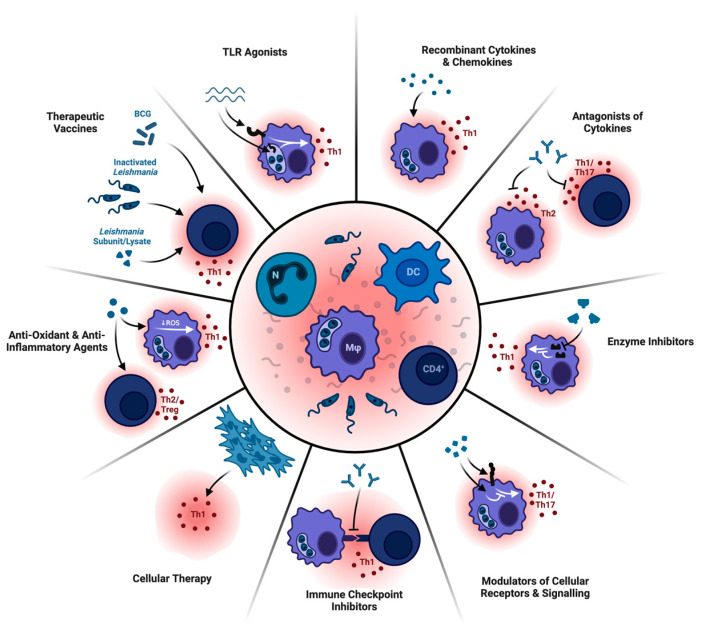
Landscape of potential immunotherapeutic approaches to treat CL. Depending on the form of CL, immunotherapies aim to promote parasitic clearance and/or disease resolution by enhancing the Th1 response, decreasing the Th2 response, or dampening exacerbated Th1/Th17 inflammatory responses. BCG, Bacillus Calmette–Guerin; CD4^+^, CD4^+^ T cells; DC, dendritic cells; N, neutrophils; Mφ, macrophages; Th1, T helper 1 cytokines; Th2, T helper 2 cytokines; Th17, T helper 17 cytokines; TLR, toll-like receptor; Treg, regulatory T cell cytokines.

Immunotherapies encompass a variety of small molecules and biologics with immunomodulatory activity (inhibitory or stimulatory) that skew immune responses to promote parasite eradication and/or disease resolution (Figure 1) [12]. Such therapeutics may be administered alone or in combination with current antileishmanials. Given the significant toxicity associated with many antileishmanial treatments, the possibility of using immunotherapies to enhance their therapeutic effect at lower dose levels or reduced dose frequency, potentially curbing side effects, is of particular interest [12].

### 3.1. Recombinant Cytokines and Chemokines

The use of recombinant cytokines and chemokines as therapeutic interventions for CL has been studied extensively in both animal models and human studies (summarised in Table 1).

Treatment using recombinant Th1 cytokines has been used to reverse nonhealing Th2-skewed CL with mixed results. In a seminal study, the use of recombinant IFN-γ, one of the main effectors of the Th1 response, in combination with antimonial therapy, was shown to enhance IL-12 and iNOS and reduce IL-4, IL-10, and TGF-β, promoting wound healing in a murine model infected with *L. major*—although this effect was not reproducible with IFN-γ treatment alone [99]. Both systemic monotherapy and treatment with IFN-γ in combination with pentavalent antimonials have proven effective in cases of complex human CL caused by *L. donovani,* as well as in earlier studies on *L. major* and *L. tropica*, although topical formulations are ineffective regardless of causative species and disease severity [85,99,100]. IFN-γ has not been widely used for CL, and recent studies have not investigated it further, likely due to its limited effectiveness as a monotherapy, inconsistent results when used in combination therapies, and notable adverse effects [101].

Infection of susceptible mice with *L. major* has been successfully treated with recombinant IL-12—a Th1 stimulatory cytokine typically produced by monocytes/macrophages and DCs [102]. Treatment is associated with a decrease in IL-4, a significant reduction in parasite burden, and a Th1 response that can be enhanced by combined treatment with the cyclooxygenase inhibitor indomethacin [93,103]. Furthermore, when administered in combination with pentavalent antimonials, IL-12 stimulates a Th1 switch and is protective against CL relapse [92]. Interestingly, it has been reported that IL-12 treatment is not effective at reducing parasite burden or pathology caused by murine *L. mexicana* infection, highlighting the impact of the causative species on disease progression [88]. To date, no studies have explored recombinant IL-12 in human CL, although earlier ex vivo studies of PBMCs from human VL patients corroborate its ability to stimulate a Th1 response [104].

The pro-inflammatory cytokine IL-18, which acts synergistically with IL-12 to activate NK cells and induce a Th1 response, has also been suggested as a treatment for CL and studied in murine models [89,105]. However, while one study has shown that IL-18 administered in conjunction with IL-12 is protective against excessive footpad swelling induced upon *L. major* infection, more recent evidence indicates that the cytokine could be involved in susceptibility to *L. amazonensis* infection [89,105].

IL-1α is another pro-inflammatory cytokine involved in T cell differentiation. Treatment of *L. major*-infected Balb/c mice with recombinant IL-1α has proven effective in reducing lesion thickness and inducing a Th1 response during T cell priming (days 1 to 3 post-infection) [86]. However, prolonged IL-1α treatment (3 weeks) worsened disease outcomes through Th2 expansion, suggesting that IL-1α treatment may be promising for early infections but not for established CL [86,87].

Granulocyte-macrophage colony-stimulating factor (GM-CSF), a cytokine with diverse roles in phagocyte maturation and differentiation, has been used in CL treatment with mixed results. Initial protocols in phase 2/3 clinical trials in LCL patients using intralesional injections of GM-CSF in combination with antimonial therapy significantly increased healing [95]. Further studies in cases of refractory CL using topical formulations of the cytokine in combination with SSG indicated a significant decrease in lesion severity and an increase in clinical cure rates compared to antimonial treatment alone [106]. The same trend was not observed in two double-blind, randomised trials assessing the use of GM-CSF in combination with miltefosine, which reported no clinical benefit of the addition of cytokines to the regimen [96,97]. An ex vivo study of circulating immune cells collected from patients with *L. braziliensis* infection treated with this combination therapy, however, revealed an increase in CD4^+^ T cell proliferation and oxidative burst, and a decrease in parasitaemia [98]. While this suggests a reprogramming of the immune response, it may be insufficient to restore protective Th1/Th2 balance [98]. However, GM-CSF is not widely adopted as a standard treatment due to limited large-scale studies and regulatory approval processes.

CXCL10 is a chemokine that promotes the recruitment and activation of Th1, NK, B, and phagocytic cells. Treatment with recombinant CXCL10 has been shown to be effective in treating antimonial refractory CL in *L. braziliensis*-infected mice [90]. When recombinant CXCL10 is administered alone, CL lesions display a distinct inflammatory infiltrate profile comprising macrophages, lymphocytes, and granulomas, along with increased IFN-γ, IL-10, and TGF-β [90]. Treatment of experimental *L. major* infection with an engineered strain of *L. tarentolae* expressing CXCL10 significantly reduces parasite burden and parasitotoxic responses and favours a Th1 response in mice [91]. Gene therapy using an expression vector encoding CXCL-10 has yielded less pronounced results [91]. In order to use recombinant CXCL10 as immunotherapy, the clinical presentation of CL must be stratified, as this approach may pose a risk for progression to MCL [56]. Additional chemokines, including the monocyte chemoattractant protein-1 (MCP-1/CCL2) and macrophage inflammatory protein 1a (MIP-1a/CCL3), have been proposed as potential immunotherapeutic agents for leishmaniasis, although current studies have only focused on their use in the treatment of VL [107].

While recombinant cytokines and chemokines may be promising new treatments for some forms of CL, their short half-life, toxicity, pleiotropic activity, and manufacturing cost may present challenges to their widespread use [108].

### 3.2. Antagonists of Cytokines

Antagonising anti-inflammatory Th2 or pro-inflammatory Th1 cytokines/signalling has yielded promising results in murine models of *L. major* and *L. (Viannia)* infection (summarised in Table 2).

In the chronic phase of CL, the blockade of IL-10R with mAbs promoted a sterile cure of C57BL/6 mice infected with *L. major*, potentially reducing the risk of latency and reactivation [109]. Furthermore, studies of circulatory T cells isolated from CL patients infected with *L. braziliensis* have indicated that treatment with anti-IL-10 mAbs can stimulate IFN-γ production and can abrogate pathogenic immunotolerance caused by intralesional Tregs [110]. A similar ex vivo study evaluating the partial blockade of IL-10 in PBMCs from *L. braziliensis*-infected CL patients revealed a decrease in IL-10 and IL-4 production to basal levels, though limited effects on TNF-α production indicate a remaining risk for mucosal spread and hyperinflammation [111].

Inhibition of the production of IL-17A, a cytokine associated with the development of mucosal lesions, with digoxin or SR1001 prevented LRV1-dependent metastasis of *L. guyanensis* [55]. While no studies address the use of IL-13 or TGF-β antagonists in the context of CL, both have proven effective at reducing tissue parasite burden in a murine VL model [113].

IL-1α and IL-1β are pro-inflammatory cytokines primarily secreted by macrophages that signal through IL-1R and contribute to the inflammatory environment during *Leishmania* infection. These cytokines are involved in the activation and polarisation of T cells, particularly promoting Th1 and Th17 responses, which are essential for initiating effective immune responses against *Leishmania* parasites [21,114]. However, their excessive production can lead to hyperinflammatory conditions, exacerbating tissue damage and worsening disease outcomes [21]. The pharmacological blockade of IL-1 signalling has indicated that mAb therapy with anti-IL-1β is effective at treating skin lesions in mice infected with *L. braziliensis* [112]. Similarly, treatment with a recombinant IL-1R antagonist increased survival and decreased lesion severity in both *L. braziliensis*- and *L. major*-infected mice [112,115]. Interestingly, treatment with anti-IL-1α mAbs had no effect in this model. This is of significant interest, as commercially available immunotherapies, such as canakinumab (anti-IL-1β neutralising monoclonal antibody) and anakinra (recombinant IL-1R antagonist), could potentially be repurposed for some forms of CL, although no formal studies have explored this to date [116]. Since IL-1β is produced following activation of the NLRP3 inflammasome, some studies have been performed in the same murine models using small molecule inhibitors of NLRP3, showing a similar beneficial effect in reducing the development of skin lesions (further discussed in Section 3.5).

While antagonism of anti-inflammatory cytokines may benefit Th2-skewed CL and the blockade of pro-inflammatory cytokines could prove beneficial to Th1-biased CL, the use of mAbs presents similar challenges to the use of recombinant cytokines, including a limited production capacity, given the need for specialised facilities and expertise, high cost of production, and the risk of side effects due to their pleiotropic nature [108].

### 3.3. Immune Checkpoint Inhibitors

Immune checkpoints are cellular receptors with stimulatory or inhibitory activity that play a key role in regulating the function of T cells. Thus, immune checkpoint inhibitors may provide a promising avenue for treating CL (summarised in Table 3) [117,118].

The blockade of the ICOS-B7RP-1 costimulatory interaction using an anti-B7RP-1 mAb has been shown to suppress the production of IL-4, IL-5, and IL-10 secretion by lymph node cells, and reduce the severity of murine CL lesions [119]. The PD-1/PD-L1 interaction, known for inhibiting T cell functions and widely targeted in the treatment of various types of cancer, has been investigated in the context of CL [120]. Treatment with mAbs targeting PD-1 and PD-L1 but not PD-L2 have been shown to increase IFN-γ production by T cells in *L. amazonensis*-infected mice, with anti-PD-1 specifically reducing IL-4 and TGF-β production [120]. Notably, treated mice had lower parasite loads but also potential markers for hyperinflammation [120]. Given evidence of high levels of PD-1 in PKDL patients, such therapies may be beneficial after treatment for VL [81]. Furthermore, the repurposing of PD-1/PD-L1 inhibitors for CL treatment is of interest, considering the existing expertise surrounding their use, although notable side effects due to their pleiotropic nature limit their clinical application [120].

Exploration of the role of the OX40/OX40L interaction, crucial in balancing effector and regulatory T cells, has yielded conflicting results in different models of CL [123]. An OX40L antagonist has been shown to suppress the development of a Th2 response in a murine CL model caused by *L. major*, enabling antileishmanial immune function and reducing pathology [121]. However, contradicting studies in VL suggest that an OX40L agonist could promote Th1 polarisation [124].

Cytotoxic T lymphocyte attenuator 4 (CTLA4 or CD152) is a well-characterized co-inhibitory receptor that has long been recognised for its constitutive expression in intracellular vesicles within Foxp3+ Tregs, as well as in conventional T and B cells during immune activation [117]. Some studies have suggested that treatment with an anti-CTLA4 mAb promotes Th2 polarisation and pathogenesis in a murine model of LCL [122,125], which could be advantageous in Th1-dominant MCL.

Earlier studies demonstrated that targeting of CD86 with mAbs reduces Th2 cytokine production and parasite burden in *L. major*-infected mice, while blocking CD80 has no therapeutic effect against CL [126]. However, no recent studies have validated these findings. Other immune checkpoints, such as CD40L-CD40, B7-CD28, and GITR, have mainly been studied in VL [127,128]. Silva and Stebut [117] have also proposed LIGHT, 2B4, and TIM-3 as potential checkpoints to be investigated for CL, although their role in CD4^+^ T cells—whether indicating activation or exhaustion—remains unclear [117]. Given the potential of these pathways to contribute both to pathology and disease resolution, further studies are required to (i) assess mechanisms underlying immune checkpoints in CL, (ii) address the translational implications of immune checkpoint inhibitors in humans, and (iii) identify the target populations for such therapeutic interventions.

### 3.4. TLR Agonists

Toll-like receptors (TLRs) are innate immune receptors that recognise microbial motifs known as pathogen-associated molecular patterns (PAMPs), and which play a pivotal role as a primary defence mechanism against microbial infections [129]. Given their significance in immune regulation, TLR agonists also represent a class of immunotherapies that could be harnessed to treat CL (summarised in Table 4).

Upon TLR activation, multiple immune cascades are triggered, culminating in pro-inflammatory cytokine production, antigen-presenting cell activation, and T-cell differentiation [129]. This activation occurs through the interaction between TLRs and various microbial components acting as potent agonists—an interaction that can be replicated with synthetic or purified molecules [129].

Pam3Cys is a synthetic triacylated lipopeptide that activates TLR2. In *L major*-infected mice, treatment with this molecule reduced lesion size and severity, which was associated with the development of both Th1 and Th17 responses [130]. However, the TLR2 agonist Z-100, a *Mycobacterium tuberculosis*-derived polysaccharide, which was effective against *L. amazonensis* in vitro, was ineffective as a monotherapy and did not provide additive therapeutic effects when co-administered with MA in a murine model of *L. amazonensis* infection [131].

TLR4 primarily recognises lipopolysaccharide, a component of the cell wall of gram-negative bacteria that results in the production of pro-inflammatory cytokines and type I IFNs [129]. Treatment with ONO-4007, a synthetic lipid A analogue and agonist of TLR4, significantly suppresses the development of CL in a murine model of *L. amazonensis* infection [132,133].

TLR7 is an endosomal TLR that recognises single-stranded RNA, and its stimulation initiates signalling pathways leading predominantly to the production of type I IFNs, including IFN-α [129]. In murine models of *L. major* infection, topical treatment with the TLR7 agonist imiquimod has been shown to significantly reduce lesion size, severity, and parasite burden, and to induce iNOS synthesis in macrophages [134,135]. While imiquimod has been efficacious as a monotherapy in animal models, phase 2 clinical studies in humans have shown no therapeutic benefit when used alone [136,137]. However, imiquimod demonstrates enhanced effectiveness when combined with SSG or paromomycin in *L. major*-infected Balb/c mice [135,148]. In phase 2/3 clinical trials, its combination with pentavalent antimonials has resulted in increased cure rates, faster treatment, and reduced scarring in cases of *L. peruviana* and *L. braziliensis* infections [143,144,145,146,147,149]. Combination therapy has also proven effective in treatment-refractory cases, highlighting the importance of stratified therapeutic approaches for cutaneous leishmaniasis [144]. Additionally, imiquimod combined with itraconazole or dapsone has shown moderate success in an early-phase clinical trial, with responsiveness rates of 56% and 70%, respectively, in human cohorts [137].

TLR9 is an endosomal TLR that recognises unmethylated CpG DNA motifs, which are highly abundant in microbial DNA. TLR9 activation results in the induction of a robust Th1 response [129]. CpG oligodeoxynucleotides (CpG-ODNs) are short synthetic single-stranded DNA molecules containing unmethylated ODNs, which activate TLR9 to induce Th1 immune responses [150,151]. CpG-ODNs consist of three main types of molecules—type A, type B, and type C—which differ in their sequence/structure and biological activity. Type A CpG-ODNs predominantly induce the production of type I IFNs but are weak inducers of pro-inflammatory cytokines; in contrast, type B CpG-ODNs preferentially induce the production of pro-inflammatory cytokines but weakly stimulate the production of type I IFNs. Type C CpG-ODNs are strong inducers of both type I IFNs and pro-inflammatory cytokines [150,151]. Treatment with type A CpG-ODN, also known as CpG-ODN-D, alone or in combination with pentavalent antimonials, has been found to be effective in various non-human primate (NHP) models of CL. In rhesus macaques (*Macaca mulatta*) and cynomolgus macaques (*Macaca fascicularis*) intradermally challenged with *L. major*, CpG-ODN-D led to the induction of IFN-α as well as IFN-γ, decreased re-epithelisation time, and smaller lesion size [139,141]. This effect was even more apparent when CpG-ODN-D was used both prophylactically and therapeutically in rhesus macaques, with one dose administered three days before *L. major* or *L. amazonensis* infection and another dose administered three days after infection [139,140]. Notably, this treatment protocol was found to reduce clinical severity and parasite burden in simian immunodeficiency virus (SIV)-infected rhesus macaques challenged with *L. major*, indicating a potential role for CpG-ODN-D in cases of human retroviral co-infection [140].

In addition, treatment with empty bacterial pcDNA3 plasmids has been reported to induce Th1 responses, downregulate Th2 mediators, and decrease lesion severity in a murine CL model of *L. major* infection [152]. While the exact underlying mechanism remains unclear, it has been hypothesised to be driven by TLR9 activation, as the pcDNA3 plasmids are rich in CpG motifs. Thus, CpG motifs, particularly type A/D CpG ODNs, have important immunotherapeutic potential, with additional research indicating their use as immunoadjuvants in live *Leishmania* vaccines [153]. Phase 2/3 clinical trials are currently underway to assess the use of CpG-ODN-D35 in human CL [154].

Although many TLR agonists induce the production of type I IFNs, often associated with MCL and DL pathology, these cytokines are involved in initial immune responses and can promote control of *Leishmania* infection [155]. For example, in BALB/c mice, type I IFNs were essential for CpG-induced protection but were dispensable for the spontaneous resolution of *L. major* infection [155]. This dual role suggests that while type I IFNs can enhance immune defences, their overproduction or prolonged activity may drive chronic inflammation and tissue damage, leading to exacerbated disease outcomes. As such, reconciling the favourable and unfavourable effects of type I IFNs remains critical for optimising therapeutic approaches in these conditions.

Further, the pharmacological blockade of TLR2 and TLR4 with mAbs has also emerged as a potential therapeutic approach in CL patients infected with *L. braziliensis*, who are at risk of developing hyperinflammatory MCL [156]. In an ex vivo study using PBMCs from these patients, antagonism of these TLRs has been shown to mitigate hyperinflammatory responses, leading to a decrease in parasitaemia and production of NO, IL-1β, TNF-α, and CXCL9 [156]. Moreover, while TLR3 has yet to be explored as a therapeutic target against CL, its implication in LRV1-mediated hyperinflammation indicates the potential use of a TLR3 antagonist in mitigating progression to MCL [65].

The widespread use of TLR agonists as adjuvants in immunotherapies and immunisations facilitates their use for the potential treatment of CL.

### 3.5. Modulators of Cellular Receptors and Signalling

Targeting other cellular receptors and downstream signalling cascades to induce a host response is a potential way to achieve an antileishmanial response and promote the resolution of CL (summarised in Table 5).

The aryl hydrocarbon receptor (AhR) is a ligand-activated transcription factor that is involved in T cell differentiation [157]. A single local injection of the AhR agonist ITE was reported to induce pro-inflammatory cytokines and reduce lesion severity and parasite burdens in early time points of murine *L. major* infection [157].

The NLRP3 inflammasome is a cytosolic multimeric protein complex, which is a member of the innate immune family of pattern recognition receptors (that also includes TLRs). NLRP3 is activated upon recognition of various damage-associated molecular patterns and PAMPs, including those from *Leishmania,* to trigger inflammatory immune responses through the production of the pro-inflammatory cytokines IL-1β and IL-18 [180]. In the context of *Leishmania* infection, NLRP3 is essential for amplifying the Th1 response, thereby controlling parasitic infections [114]. However, the activation of the NLRP3 inflammasome also contributes to the production of IL-1β, which, when unregulated, can drive excessive inflammation, potentially leading to chronic tissue damage and poor clinical outcomes [181]. The balance between the beneficial and detrimental effects of the NLRP3/IL-1β axis is influenced by various factors, including the species of *Leishmania*, the stage of infection, and the host’s genetic background [69,70,84,182,183]. Given the dual roles of NLRP3/IL-1β in both promoting immune defence and driving pathological inflammation, therapeutic strategies that modulate the NLRP3/IL-1β axis must carefully balance these opposing effects. As described above in Section 3.2, anti-IL-1β mAbs had a beneficial effect in resolving CL in mice infected with *L. braziliensis*. Consistent with this, NLRP3 inflammasome inhibitors, such as glyburide (also known as glibenclamide) and MCC950, substantially decreased pathology when administered to mice infected with *L. major* [112]. Similarly, glyburide increases IFN-γ production, and decreases TNF-α, IL-4, and IL-10 expression, resulting in disease resolution in another murine model of *L. major* infection [159]. In a model of *L. braziliensis* infection in RAG mice reconstituted with CD8 cells (RAG + CD8)—developed to study the contribution of CD8^+^ cells to immune responses—both glyburide and MCC950 treatment were found to reduce lesion size but not parasite burden [160]. Using skin biopsies from *L. braziliensis*-infected patients, ex vivo treatment with glyburide significantly decreased IL-1β, IL-17, and TNF release while leaving IFN-γ, IL-6, and IL-10 levels unchanged in these tissues [160]. Furthermore, administration of glibenclamide in combination with pentavalent antimonials resulted in a reduction in lesion progression, even in cases of drug resistance [158]. Although it has been proposed as a therapeutic intervention to mitigate hyperinflammatory CL, earlier reports suggesting a direct correlation between NLRP3 subversion and MCL severity present conflicting results [84]. However, this may be explained by the variable roles of the inflammasome during the progression of the disease and divergent responses to different species of *Leishmania* [84].

Antagonists of NF-κB, including the flavonoid quercetin, have also shown the capacity to decrease lesion severity and parasite burden while increasing survival in various murine models [161,162,163,164]. Studies in Syrian hamsters indicated that prolonged regimens of oral quercetin are effective at decreasing lesion severity and parasite burden [165]. While topical administration has moderate efficacy, formulations containing nanosomal quercetin have proven much more effective both topically and orally, indicating the importance of drug bioavailability and the delivery system [161,162,163,164].

Chitin, a homopolymer of N-acetyl glucosamine, is present in the cell walls of fungi and the exoskeletons of insects and other arthropods [166]. It binds to and stimulates the chitinase 3-like-1 (CHI3L1) PRR and contributes to TLR signalling. Its deacetylated form, chitosan, can also stimulate Dectin-1 [166]. In a murine model of CL caused by *L. major*, both chitin and chitosan significantly reduced lesion swelling, and chitin alone induced the production of TNF-α and IL-10 [166]. Chitosan nanoparticles have also been used as a drug-delivery system for amphotericin B, enabling complete wound healing and parasite inhibition, and decreasing the number of inflammatory granulomas [168,172,173,176]. Furthermore, the use of chitosan nanoparticles has been shown to significantly reduce toxicity induced by amphotericin B treatment [174]. These nanoparticles have also been successfully used for the delivery of betulinic acid, b-lapachone, and nitric oxide, with varying levels of success [176,178,179]. Topical use of chitosan treatments has been shown to have moderate efficacy compared to systemic treatment in a murine model [167,172,178]. In a pilot clinical study (phase 1) of chitosan-based dressing of CL wounds, there was significant improvement or clinical cure in all patients, although no control group was included [169].

Human neutrophil peptide 1 (HNP1), an antimicrobial peptide produced by neutrophils, has the ability to interact with multiple receptors, including TLR4, thereby influencing downstream signalling events [170]. Treatment with both synthetic folded HNP1 and pcDNA HNP1 gene therapy has proven effective in inducing Th1 polarisation and limiting parasite burden in a murine CL model [170]. Additionally, live treatment of mice with *L. tarentolae* expressing HNP1 resulted in a Th1-polarised response, leading to the resolution of CL [171].

### 3.6. Enzyme Inhibitors

The targeted inhibition of specific host enzymes has emerged as an immunotherapeutic strategy for CL (summarised in Table 6). These drugs hold promise due to their cost-effectiveness and clinical approval for other conditions.

Kinase inhibitors have shown promise in modulating cellular signalling cascades. Mechanistic target of rapamycin (mTOR) inhibitors can be used to alter inflammatory signalling pathways. The mTOR pathway is involved in the activation and function of immune cells, the regulation of autophagy, and numerous metabolic processes [184,185]. Targeting mTOR signalling with inhibitors, including rapamycin and its analogue GSK-2126458, has been shown to induce a Th1 response and dramatically reduce footpad swelling and parasite load in the draining lymph node of *L. major* and *L. tropica*-infected mice [184,185]. This was not replicated with the rapamycin analogue KU-0063794, and combination therapy with antimonials or amphotericin B added no therapeutic benefit to rapamycin treatment [184,185]. Phosphoinositide 3-kinase (PI3K) is a key signalling enzyme involved in T cell differentiation, macrophage activation, and regulation of autophagy and phagocytosis [186]. Treatment of a murine CL model with AS-605240, an inhibitor of PI3Kγ, effectively decreases lesion size and parasite load, resulting in a therapeutic response that is comparable to that of antimonial therapy; combination therapy with antimonials provides an additive therapeutic effect [186]. Additional PI3K inhibitors, including idelalisib (CAL-101) and AS101, have been tested in VL, yielding promising results which may translate to CL [205].

The β-carboline alkaloid harmine is a multikinase inhibitor known to inhibit monoamine oxidase A and dual-specificity tyrosine-regulated kinases, particularly DYRK1A, CLK1, and CLK4 [187]. In a murine model of infection by multidrug-resistant *L. major*, synthetic harmine (ACB1801) monotherapy resulted in decreased cutaneous lesion severity and parasite burden accompanied by increased TNF-α- and IFN-γ-producing CD4^+^ T cells and decreased immunosuppressive FOXP3+ T cells, resulting in comparable efficacy to amphotericin B [187]. Furthermore, treatment with ACB1801 downregulated AhR, highlighting the different levels of modulation at which these pathways can be targeted. Combination treatment with ITE, for example, may enable more robust inhibition of this signalling pathway and downstream immunosuppression.

The JAK/STAT pathway is fundamental to IFN-γ signalling and macrophage activation and can be directly modulated by *Leishmania* parasites to increase survival and virulence [188]. The JAK1/3 inhibitor tofacitinib has previously been shown to reduce CD8^+^ T cell cytotoxicity, resulting in protection against the development of severe lesions in murine models [188]. Through IL-15 blockade, tofacitinib attenuates granzyme B expression by CD4^+^ T cells but does not diminish the Th1 profile [188,206]. A clinical trial assessing the efficacy of tofacitinib in combination with MA is currently in progress [207].

Ibrutinib, a Bruton tyrosine kinase inhibitor that has significant anti-interleukin-2-inducible kinase activity, is effective at skewing the immune response towards Th1 and reducing lesion size and parasite burden in a murine CL model [189]. Imatinib, a small molecule inhibitor targeting tyrosine kinases, exerts a similar therapeutic effect on CL lesions, although to a lesser extent than classical treatments such as amphotericin B [190,191].

Elevated arginase activity has been correlated with non-healing CL, contributing to local depletion of L-arginine [208]. This depletion hampers the proliferative capacity of intralesional T cells, impeding the effective clearance of infection, and is directly associated with the development of ADCL [208]. Administration of the arginase antagonist nor-NOHA in a murine CL model results in a reduction in parasite load, although the impact on lesion size is not statistically significant [192]. The treatment’s effectiveness is attributed to the induction of parasite killing through NO production [193]. Notably, the inhibitory effect on lesion severity is observed primarily at early time points post-infection, with the protective influence diminishing over the course of the disease [194]. Nor-NOHA also exhibits direct antileishmanial activity by inhibiting leishmanial arginase [209]. Additionally, supplementation of L-arginine, circumventing depletion of the substrate, has been shown to restore intralesional T cell responses in vivo, resulting in reduced lesion severity and parasite burden, offering a potentially cost-effective and accessible therapeutic intervention for CL [192,195].

The phosphodiesterase inhibitor pentoxifylline inhibits TNF-α and other pro-inflammatory mediators in a dose-dependent manner [196]. In a murine CL model, pentoxifylline treatment reduces parasitaemia and lesion severity and activates macrophages, contributing to its efficacy [196]. Despite showing initial promise, phase 2 trials have indicated no therapeutic benefit from adding pentoxifylline to antimonial or miltefosine therapy for LCL, while there is the potential for additional adverse effects when combining treatments [200,201,203]. However, pentoxifylline, in conjunction with pentavalent antimony, has had success in treating refractory MCL, leading to a reduced treatment duration in phase 2 trials and indicating potential utility in complex hyperinflammatory CL [202,204]. Notably, pentoxifylline is included in PAHO recommendations for the treatment of MCL in the Americas, underscoring the translational potential of immunotherapies in clinical practice [9].

Statins, specifically HMG-CoA inhibitors, are recognised for their well-documented anti-inflammatory and immunomodulatory properties [197]. Treatment with pravastatin has improved survival and reduced weight loss in various murine and Syrian hamster models infected with *L. amazonensis* [197]. This effect is attributed to the modulation of macrophage function, characterised by increased NO production, reduced phagocytosis, and limited TNF production [198]. Similarly, topical formulations of simvastatin have reduced tissue damage and parasite burden in lesions caused by murine *L. major* infection [199].

### 3.7. Anti-Inflammatory and Antioxidant Agents

Anti-inflammatory and antioxidant agents are particularly appealing for the management of hyperinflammatory presentations of CL, given their widespread use and accessibility (summarised in Table 7).

Acetylsalicylic acid (ASA), a non-steroidal anti-inflammatory drug, has the ability to stimulate the production of NO by macrophages [210]. In a murine CL model, oral ASA treatment resulted in a reduction in lesion size and amastigote proliferation [210]. This finding is of particular interest due to the widespread use of ASA as an over-the-counter painkiller, and the associated low cost of production, shelf-stability, and ease of procurement. In topical formulations, the administration of ASA, salicylic, or ascorbic acid with potassium nitrite in NO-generating ointment proved ineffective in treating both murine *L. tropica* lesions and a phase 2 clinical study for human CL [216].

Gentisic acid, a derivative of salicylic acid, has been investigated for its capacity to inhibit the production of pro-inflammatory cytokines and prostaglandins, as well as its protective effects against oxidative stress [211]. Treatment of *L. amazonensis*-infected BALB/c mice with gentisic acid resulted in a decrease in both lesion size and parasite load [211]. Although equally effective in decreasing CL lesion size compared to gentisic acid, it demonstrated limited impact on parasitaemia [211]. P-coumaric acid, another phenolic compound, has been reported to enhance the activity of superoxide dismutase and catalase while inhibiting various inflammatory pathways [211].

Plant-derived phenolic compounds including gallic and ellagic acids have shown anti-inflammatory effects in *Leishmania* macrophage infection in vitro, suggesting a potential therapeutic benefit for Th1-skewed CL [217]. Treatment with both compounds has proven effective in activating a healing immune response, resulting in reduced lesion size and severity in a murine CL model, although no studies have assessed the effect in complex disease presentations [212]. The therapeutic effect is further enhanced when either compound is administered in combination with amphotericin B [212]. Ursolic acid has been reported to induce Treg activation and IL-10 production while inhibiting the production of the pro-inflammatory cytokines IL-6, TNF-α, and IL-1β [213]. When administered topically, this compound reduces lesion progression in a chronic CL infection model caused by *L. amazonensis* [213].

The isoquinoline alkaloid berberine chloride has diverse anti-inflammatory and antioxidant functions, including the inhibition of cyclooxygenase-2 (COX-2), lipoxygenase (LOX), and NF-κB activation, as well as scavenging ROS [214]. Topical berberine treatment in experimental CL caused by *L. major* was shown to reduce inflammatory cell infiltration into lesions, resulting in decreased lesion size and parasite load [214].

The heterosidic steroids solamargine and solasonine, extracted from the Brazilian plant *Solanum lycocarpum*, reduce lesion size and severity in a murine CL model when applied topically [215]. Although potentially useful for remote communities affected by CL, studies assessing the use of entire plant extracts have given conflicting results, due to variability in composition between preparations.

Natural acids and phenolic compounds may be of interest for CL drug development, due to minimal technical requirements for their production and isolation, and the general effectiveness of localised topical administration, facilitating drug administration and minimising side effects.

### 3.8. Cell Therapy

Cell therapy has been explored as a therapeutic intervention for CL in animal models with limited success (summarised in Table 8).

In a 2017 study, bone marrow mesenchymal stromal cells (MSCs) were administered to *L. amazonensis*-infected mice via intralesional or intravenous injection [218]. While no difference in lesion size or progression was reported, regardless of administration route, mice that received intravenous MSCs had higher parasite loads—indicating a potentially deleterious effect of the treatment [218]. In contrast, another study reported that MSCs alone can limit lesion size, and that combination therapy with pentavalent antimonials is particularly effective at decreasing parasitaemia [219].

Bone marrow dendritic cells (BM-DCs) have also been studied as a potential therapeutic intervention against CL, particularly in chronic CL caused by *L. amazonensis* [220]. However, although the transfer of antigen-pulsed BM-DCs to mice along with IL-12 enhanced the Th1 response, it failed to ameliorate clinical symptoms [220].

In addition to the limited therapeutic efficacy of cellular therapy, these treatment modalities are likely not feasible in remote CL-endemic regions, and the clinical benefit may be restricted to a few treatment-unresponsive cases.

### 3.9. Therapeutic Vaccines

Therapeutic vaccines, combining inactivated *Leishmania* or leishmanial antigen preparations with immunoadjuvants, present a novel approach for treating CL by stimulating an antiparasitic immune response (summarised in Table 9).

Heat-killed or inactivated *Leishmania* parasites have been successfully used in therapeutic vaccines against CL. For instance, heat-killed *L. amazonensis* achieved a remarkable 98.1% cure rate in a human cohort study (phase 3), although this required an extended treatment duration compared to conventional chemotherapy [221]. Combining the inactivated parasite with antimonial therapy resulted in successful treatment with reduced antimony doses, decreasing the risk of secondary toxicity [221,234].

Leishvacin©, a vaccine containing inactivated *L. amazonensis*, *L. venezuelensis*, *L. braziliensis*, and *L. chagasi* amastigotes, was developed to address the geographical co-localisation of various species of *Leishmania*, particularly in Central and South America [222]. When administered alone in phase 2/3 trials, the vaccine has been shown to effectively cure CL in human subjects and reduce remission and secondary infections [222]. While not broadly approved globally, it has been utilised to treat human CL in some regions of Brazil [222]. No additional therapeutic effect is observed when administered in combination with pentavalent antimonials, though PBMCs from patients treated with immunochemotherapy showed enhanced lymphoproliferative response in ex vivo studies [235].

Use of the immunoadjuvant Bacillus Calmette–Guerin (BCG), a non-specific stimulant of TLR2, TLR4, and other PRRs, has proven effective in combination with killed/inactivated *Leishmania* vaccines. The live bacterium, conventionally used as an attenuated vaccine against tuberculosis, has been shown to stimulate a potent Th1 response and result in full clinical recovery when combined with *L. amazonensis*, benefiting both LCL and MCL patients [223,224]. Notably, in a phase 2 trial and an earlier study, BCG formulations with *L. braziliensis* had a high healing rate of 95.7%, emphasising potential therapeutic efficacy for complex CL, such as cases of MCL and ADCL [225,226]. Furthermore, most treatment failures and adverse effects in a retrospective cohort study were reported in LCL and cases without mucosal involvement [225,226].

In a patient with treatment-resistant DL, inactivated *L. amazonensis*/*L. braziliensis* was administered with BCG and antimonial therapy, resulting in lesion resolution, although re-activation occurred following completion of treatment [236]. Moreover, immunochemotherapy employing a combination of heat-killed *L. major*, BCG, and antimonial therapy emerged as a potent strategy against persistent PKDL in a phase 2 trial [237,239]. This success is attributed to an increase in IFN-γ levels, offering a valuable therapeutic approach to addressing persistent cases of CL [237,239].

While several studies have explored the therapeutic use of BCG alone in cases of human CL, resulting cure rates ranged from 38 to 42%, suggesting that it is more effective as an adjuvant to other therapies [221]. Combination of BCG with low-dose SSG, in the absence of *Leishmania*/*Leishmania* antigen, has proven extremely successful at treating human CL, with cure rates comparable to that of full courses of antimonial therapy in a phase 3 trial [221].

Furthermore, studies of vaccines comprising soluble *Leishmania* antigen (SLA; produced by lysing whole parasites) and immunoadjuvants have reported promising results. SLA, combined with the TLR2 agonists Pam3Cys and the TLR8 agonist resiquimod (R848), was shown to control CL lesions and parasite loads by inducing IFN-γ and NO production in a murine model [227]. TLR synergy has also been harnessed using the TLR4 agonist ONO-4007, a synthetic lipid A analogue, which has been shown to enhance SLA-based vaccine efficacy, contributing to the successful treatment of refractory CL [133]. However, while the production of SLA vaccines requires minimal technical equipment, making it a cost-effective treatment strategy, parasitic developmental stage- and condition-dependent variability in SLA composition may present a challenge for consistent treatment results in real-life settings.

Second-generation vaccines, featuring recombinant or purified antigens either alone or in adjuvant formulations, provide a much more consistent approach and have had some success. A vaccine containing recombinant TSA, LmSTI1, LeIF, and HSP83, administered in combination with GM-CSF, has effectively treated patients with treatment-refractory MCL and prevented disease relapse in a phase 2 trial [228,229]. Combination treatment using pentavalent antimonials and the Leish-F1 vaccine, comprising polyprotein combining TSA, LmSTI1, and LeIF fused in tandem, along with the adjuvant MPL, achieved an 80% clinical cure rate in LCL patients in a preliminary (phase 1) study [238]. A modified version of the Leish-F1 protein, L110f, resulted in strong Th1 responses and clinical cures when combined with lipid A (monophosphoryl or glucopyranosyl) and the TLR9 agonist CpG-ODN-B [230].

The *L. donovani* nucleoside hydrolase NH36, administered with saponin, is therapeutic in murine CL, increasing IgG2b antibodies and levels of IFN-γ and TNF-α without reducing IL-10 [231]. This finding highlights cross-immunity amongst various species of *Leishmania*, likely due to the high levels of homology between leishmanial antigens.

Furthermore, a DNA vaccine encoding the parasitic surface antigen GP46 has been shown to induce a Th1 response and significantly reduce lesion size in a murine CL model [232]. The use of nucleic-acid-based vaccines for leishmaniasis is an interesting avenue of research due to the low cost of production and relative stability of the materials, facilitating distribution and administration [232]. In addition, mRNA vaccine technology, which has recently demonstrated widespread success in immunisation campaigns against SARS-CoV2, could be harnessed to express such leishmanial antigens.

Finally, ChAd63-KH, a third-generation vaccine comprising a replication-defective simian adenovirus vector expressing leishmanial proteins KMP-11 and HASPB, achieved a 30% cure and 20% clinical improvement in Sudanese PKDL patients in an early-phase (phase 2a) trial, with minimal adverse effects and potent innate and cell-mediated immune responses [233].

In summary, while most studies on leishmanial vaccines address their protective capacity, the repurposing of such formulations to induce clinical cure following the establishment of acute and chronic CL is an interesting avenue for future research. However, despite potential promise, no CL vaccines have been approved for prophylactic or therapeutic use in humans to date.

## 4. Discussion and Future Directions

Developing novel therapeutic interventions for acute and complex CL should be a priority, given the already significant burden of this disease throughout low- and middle-income countries and the anticipated expansion of endemic areas due to climate change [8]. Furthermore, current CL treatments are associated with important toxicity, and there is a risk of emerging drug resistance [240,241]. While local treatments can mitigate toxicity, especially in pregnant women or in people with HIV co-infection [71], topical treatments may not be suitable for lesions prone to dissemination or mucosal involvement. Second-line treatments, including paromomycin, miltefosine, pentamidine, and amphotericin B deoxycholate—which is particularly effective for antimonial-unresponsive CL—are similarly accompanied by severe adverse effects [11]. To reduce toxicity, a liposomal formulation of amphotericin B has been developed, with similar efficacy to the original drug, and has successfully been used in complex cases of CL including MCL, DL, LR, and PKDL [11]. This underscores the importance of bioavailability and targeted delivery of antileishmanial drugs, given that *Leishmania* is an intracellular pathogen. However, the high cost of treatment regimens that include liposomal amphotericin B limits access, requiring the use of less-effective and more toxic alternatives [11].

Furthermore, combining conventional therapies may enhance effectiveness, as is the case of intralesional antimonial administration along with cryotherapy [11]. Combination therapy is an important tool for reducing the required dose of conventional antileishmanial therapeutics, mitigating toxicity, and delaying the emergence of drug resistance [242]. As CL results from both parasitic infectivity and host response, adjuvant immunotherapies may be particularly efficacious when administered with conventional chemotherapies.

Immunotherapies for CL would be particularly beneficial for remote endemic areas throughout the tropics and subtropics; therefore, it is important to consider the cost of production and temperature stability of the therapeutic materials when planning the implementation of immunotherapies in treatment protocols. Consequently, biologics, including mAbs and recombinant cytokines, as well as cellular therapies, likely display limited potential, while small molecule inhibitors, TLR agonists, and DNA-based therapeutic vaccines should be explored further [156].

TLR9 agonist CpG-ODNs, particularly type D/A, have shown considerable efficacy in enhancing the immune response against *Leishmania* infection in NHPs. Their ability to boost both the innate and adaptive immune responses makes them suitable candidates for both prophylactic and therapeutic interventions, with types K/B and D/A CpG-ODNs being successful adjuvants in heat-killed and live *Leishmania* vaccines [153,243]. Importantly, CpG-ODN-D35 has shown promise both as a monotherapy and in combination with pentavalent antimonials for established CL in various NHP models and is a current focus of research efforts. CpG-ODNs can be designed to be cost-effective and stable at varying temperatures, which is essential for deployment in resource-limited settings. Additionally, their relatively small size and straightforward production process make them feasible for large-scale use in endemic regions.

As the pathogenesis of leishmaniasis is intricately shaped by the dynamic interplay between the host and the pathogen, diverse forms of acute and complex CL exhibit heterogeneous immunopathogenesis, adding a layer of complexity to treatment. For example, while effective against CL, pentamidine cures lesions at a slower rate than antimonial therapy, increasing the risk of parasitic metastasis and MCL development [74]. In terms of immunotherapies, pentoxifylline/antimony combination treatment and therapeutic vaccines with GM-CSF may be efficacious in cases of MCL, while CXCL10 should be avoided due to the increased risk of disease progression. Additionally, in vitro and animal studies suggest that inhibitors of the NLRP3/IL-1β axis may have some value as treatments for CL forms associated with exacerbated Th1 and Th17 responses, such as MCL. Furthermore, BCG/*Leishmania* vaccines have proven efficacy in MCL, ADCL, and DL, potentially circumventing the need for speciation prior to treatment. ADCL may also be treated by targeting arginase with Nor-NOHA or with L-arginine supplementation, although further studies should investigate these interventions. PKDL may require distinct treatments, as causative species diverge from CL-specific *Leishmania* [81]. Thus far, several candidate vaccines for VL have shown promise for therapeutic use in PKDL [233]. The efficacy of immunotherapeutic interventions for complex CL is dependent on the causative species of *Leishmania*, the stage of disease, and the immune status of the patient. Addressing this challenge necessitates a profound understanding of the intricate pathways involved, underscoring the importance of basic research to inform drug discovery. Moreover, while parasite speciation may be crucial to the informed determination of treatment strategies, point-of-care biopsy, culture, and biochemical methods may not be feasible. Therefore, the development of rapid and accessible testing adapted to resource-limited settings should be a research priority.

Given the limited resources in NTD drug discovery, the repurposing of immunotherapies for cancer or autoimmune diseases and of medications with off-label immunotherapeutic effects, having already completed extensive safety trials, reduces the time required for approval and is a particularly interesting avenue for novel CL treatment. These medications include statins, glyburide, and ASA, along with immune checkpoint inhibitors for severe treatment-refractory cases. In addition, the widespread administration of the BCG vaccine as a preventative measure against tuberculosis suggests the presence both of an established supply chain and of roll-out measures of the vaccine worldwide [223,224]. It follows that a BCG-based vaccine, with the addition of recombinant leishmanial peptides or nucleic acids, could feasibly be made available as a therapeutic adjuvant or alternative to conventional chemotherapy.

Although significant work has been carried out to study the use of various classes of immunotherapies in the treatment of CL, a wide gap remains between studies performed in animal models and translational research in clinical studies. The lack of reliable animal models for complex forms of CL is an important challenge when addressing the effect of immunotherapies on abnormal immune responses characteristic of complex human leishmaniasis. Furthermore, many in vitro studies with promising results cannot be replicated in vivo, underscoring the impact of bioavailability. The use of nanosomal formulations and nanoparticles may enable targeted delivery of therapeutics, and decorating nanoparticles can provide an additional level of immune modulation to therapeutics [173,174,179]. Thus, delivery systems should be explored further for combination immunotherapies.

Additional challenges arise in clinical contexts due to a lack of a global standard framework of diagnostic criteria to differentiate between acute and complex forms of CL. Given that there are important immunological differences between various forms of CL, which may not be immediately apparent from the clinical manifestations alone, there is a risk of using the wrong immunotherapy and inducing an improper, and likely pathological, immune response. Thus, it is essential to develop a framework of diagnostic criteria that are applied consistently or to identify highly specific biomarkers for each of the disease presentations. The use of artificial intelligence for the stratification of such groups, which has previously been used for dermatoses and other skin diseases, may be an important tool for the future of CL diagnosis and treatment.

In conclusion, immunotherapies can be used to inform the immune response during acute and chronic CL to limit clinical manifestations and disease severity. While many are effective alone, administration of immunotherapies in conjunction with conventional chemotherapies has proven particularly effective, enhancing the effect of antileishmanial drugs, and limiting associated toxicity. However, although immunotherapies hold promise for the treatment of CL, further research is required to identify their optimal use, and many considerations surrounding their feasibility, accessibility, and therapeutic benefit must be addressed before their inclusion in standard treatment protocols.

## Figures and Tables

**Table 1:** Animal and clinical studies assessing the use of recombinant cytokines and chemokines for CL.

Therapy	Study Type	Study Design	Effect/Clinical Outcome	Reference
IFN-γ	Clinical case	Treatment of a 29-year-old male patient with chronic CL caused by *L. donovani* (IFN-γ 100 μg/m^2^/day SC × 6 weeks).	Decrease in parasite load and induction of wound healing.	[85]
IL-1α	Animal	Treatment of *L. major*-infected Balb/c mice (IL-1α ID 50 ng/day × 3 days).	Decrease in lesion size and increase in Th1 polarisation.	[86]
	Animal	Treatment of *L. major*-infected Balb/c mice (IL-1α ID 50 ng/day × 3 days and/or 50 ng/3 days × 3 weeks).	Increased lesion thickness and Th2 cell expansion.	[87]
IL-12	Animal	Treatment of *L. mexicana* or *L. major*-infected C57BL/6 mice (IL-12 0.2 μg/day IL × 12).	No therapeutic effect.	[88]
IL-18	Animal	Treatment of *L. major*-infected C57BL/6 mice (IL-18 1000 ng/day IP × 7 days).	No therapeutic effect.	[89]
IL-18 + IL-12	Animal	Treatment of *L. major*-infected Balb/c mice (IL-18 1000 ng/day IP × 7 days + IL-12 10 ng/day IP × 7 days).	Decrease in footpad swelling and parasite load.	[89]
CXCL10	Animal	Treatment of antimony refractory *L. braziliensis*-infected Balb/c mice (CXCL10 5 μg/kg IM × 7 days).	Control of lesion progression.	[90]
CXCL10 (*L. tarentolae)*	Animal	Treatment of *L. major*-infected Balb/c mice (2 × 10^5^/week *L. tarentolae* SC × 3 weeks).	Decrease in lesion size and parasite load and increase in Th1 response.	[91]
CXCL10 (pcDNA)	Animal	Treatment of *L. major*-infected Balb/c mice (50 μg/week CXCL10 pcDNA SC × 3 weeks).	Decrease in lesion size and parasite load and increase in Th1 response.	[91]
IL-12 + PM	Animal	Treatment of *L. major*-infected BALB/c mice (IL-12 500 ng PL × 4 + PM 5% Top × 12 days).	Clinical cure and induction of Th1 response.	[92]
IL-12 + Indo	Animal	Treatment of *L. major*-infected Balb/c mice (IL-12 0.2 μg/day IL × 6 + Indo 20 μg/mL/day PO × 18 days).	Enhancement of the therapeutic effect of IL-12.	[93]
GM-CSF +Sb	Human cohort (Ph2)	Treatment of 10 adult LCL patients in an area in which *L. braziliensis* is endemic (GM-CSF ointment 0.01% Top × 3 weeks + SSG 20 mg/kg/day IV × 20 days).	Decrease in average healing time.	[94]
	Human cohort (Ph2)	Treatment of 10 adult LCL patients (GM-CSF 200 μg/week SC × 2 weeks + SSG 20 mg/kg/day IV × 20 days).	Decrease in lesion severity and increase in clinical cure rates comparatively to SSG alone.	[95]
GM-CSF + MTF	Human cohort (Ph3)	Treatment of 50 adult LCL patients (GM-CSF ointment 0.01% Top × 28 days + 2.5 mg/kg/day MTF PO × 28 days).	Limited therapeutic effect.	[96]
	Human cohort (Ph3)	Treatment of 40 adult LCL patients (GM-CSF ointment 0.01% Top × 28 days + 2.5 mg/kg/day MTF PO × 28 days).	Limited therapeutic effect.	[97]
	Human cohort (Ph3)	Treatment of adult LCL patients (GM-CSF ointment 0.01% Top × 28 days + 2.5 mg/kg/day MTF PO × 28 days).	Increase in CD4^+^ T cells and oxidative burst.	[98]

CL, cutaneous leishmaniasis; ID, intradermal; IL, intralesional; IM, intramuscular; Indo, indomethacin; IP, intraperitoneal; IV, intravenous; LCL, localised cutaneous leishmaniasis; MTF, miltefosine; PL, perilesional; PM, paromomycin; PO, per os/oral; Sb, pentavalent antimonials; SC, subcutaneous; SSG, sodium stibogluconate; Th1/Th2, T-helper 1/T-helper 2; Top, topical. Combination immunotherapy and chemotherapy treatments are identified in grey.

**Table 2:** Animal studies assessing the use of antagonists of cytokines for CL.

Therapy	Study Type	Study Design	Effect/Clinical Outcome	Reference
Anti-IL-10R mAb	Animal	Treatment of *L. major*-infected C56BL/6 mice (anti IL-10R 0.5 mg IP × 5 weeks).	Decrease in parasite load to undetectable levels.	[109]
Anti-IL-10 mAb	Ex vivo	Treatment of T cells from patients with LCL caused by *L. guyanensis.*	Increased IFN-γ production.	[110]
	Ex vivo	Treatment of PBMCs from patients with LCL caused by *L. braziliensis.*	Decreased IL-10, IL-4, and TNF-α production.	[111]
Digoxin (IL-17A antagonist)	Animal	Treatment of LRV1-positive *L. guyanensis*-infected C57BL/6 mice (digoxin 40 μg IP × 10 weeks).	Decreased lesion size and parasite burden.	[55]
SR1001 (IL-17A antagonist)	Animal	Treatment of LRV1-positive *L. guyanensis*-infected C57BL/6 mice (SR1001 20 mg/kg IP × 10 weeks).	Decreased lesion size and parasite burden.	[55]
Anti-IL-1β mAb	Animal	Treatment of *L. braziliensis*-infected RAG^−/−^ Balb/c mice (anti-IL-1β mAb 500 μg IP × 8–12 weeks).	Decrease in lesion size but not in parasite load.	[112]
Anti-IL-1α mAb	Animal	Treatment of *L. braziliensis*-infected RAG^−/−^ Balb/c mice (anti-IL-1α mAb 500 μg IP × 8–12 weeks).	No therapeutic effect.	[112]
Anti-IL-1R mAb/Recombinant IL-1R antagonist	Animal	Treatment of *L. braziliensis*-infected RAG^−/−^ Balb/c mice (anti-IL-1R mAb 500 μg IP or IL-1Ra IP 50 mg/kg × 8–12 weeks).	Decrease in lesion size but not in parasite load.	[112]

IP, intraperitoneal; LCL, localised cutaneous leishmaniasis; LRV1, *Leishmania* RNA virus 1; mAb, monoclonal antibody; PBMCs, peripheral blood mononuclear cells.

**Table 3:** Animal studies assessing the use of immune checkpoint inhibitors for CL.

Therapy	Study Type	Study Design	Effect/Clinical Outcome	Reference
Anti-B7RP1 mAb	Animal	Treatment of *L. major*-infected Balb/c and C57BL/6 mice (anti-B7RP1 mAb 300 μg/3 days IP × 80 days).	Decrease in lesion severity, and suppression of IL-4, IL-5, and IL-10 secretion from lymph node cells.	[119]
Anti-PD-1 mAb	Animal	Treatment of *L. amazonensis*-infected Balb/c mice (anti-PD-1 mAb 200 μg/week IP × 6 weeks).	Decrease in parasite load but increase in lesion size.	[120]
Anti-PD-L1 mAb	Animal	Treatment of *L. amazonensis*-infected Balb/c mice (anti-PD-L1 mAb 200 μg/week IP × 6 weeks).	Decrease in parasite load but increase in lesion size.	[120]
Anti-PD-L2 mAb	Animal	Treatment of *L. amazonensis*-infected Balb/c mice (anti-PD-L2 mAb 200 μg/week IP × 6 weeks).	No therapeutic effect.	[120]
Anti-OX40L mAb	Animal	Treatment of *L. major*-infected Balb/c mice (anti-OX40L mAb 600 μg/week IP × 50 days).	Decrease in lesion severity and parasitaemia and increase in Th1 cytokines.	[121]
Anti-CTLA4 mAb	Animal	Treatment of *L. major*-infected C57BL/6 mice (anti-CTLA4 mAb 0.3 mg IP on D0 and D7).	Increased Th2 response and disease susceptibility.	[122]

IP, intraperitoneal; mAb, monoclonal antibody.

**Table 4:** Animal and clinical studies assessing the use of TLR agonists for CL.

Therapy	Study Type	Study Design	Effect/Clinical Outcome	Reference
Pam3Cys (TLR2 agonist)	Animal	Treatment of *L. major*-infected Balb/c mice (Pam3Cys single dose 50 μg SC on day of infection).	Decrease in lesion size and severity and promotion of Th1/Th17 responses.	[130]
Z-100 (TLR2 agonist)	Animal	Treatment of *L. amazonensis*-infected Balb/c mice (Z-100 100 mg/kg/day IP × 2 weeks).	No therapeutic effect.	[131]
ONO-4007 (TLR4 agonist)	Animal	Treatment of *L. amazonensis*-infected Balb/c mice (ONO-4007 30 mg/kg/day IP/IL).	Decrease in lesion size and severity.	[132]
	Animal	Treatment of *L. amazonensis*-infected Balb/c mice (ONO-4007 50 mg/kg/day SC × 7 doses).	Decrease in lesion size and severity, and increase in IFN-γ.	[133]
Imiquimod (TLR7 agonist)	Animal	Treatment of *L. major*-infected Balb/c mice (imiquimod 5% ointment Top × 6 doses).	Decrease in lesion size and severity.	[134]
	Animal	Treatment of *L. major*-infected Balb/c mice (imiquimod 5% ointment Top × 6 doses).	Decrease in lesion size, severity, and parasite burden.	[135]
	Human cohort (Ph2)	Treatment of 12 adult LCL patients (imiquimod 5% ointment Top × 24 doses).	No therapeutic effect.	[136]
	Human cohort (Ph2)	Treatment of 6 adult LCL patients (imiquimod 5% ointment Top × 18 doses).	No therapeutic effect.	[137]
CpG-ODN (TLR9 agonist)	Animal	Treatment of *L. panamensis*-infected Balb/c mice (CpG-ODN-B 186 ng/week PL × 2 weeks).	No therapeutic effect and increase in parasite burden.	[138]
	Animal	Treatment of *L. panamensis*-infected Balb/c mice (nanosomal CpG-ODN-B 186 ng/week PL × 2 weeks).	Decrease in lesion size and severity.	[138]
	Animal	Treatment of *L. major*-infected NHPs (single dose CpG-ODN-D/A 0.5 mg/kg ID).	Decrease in lesion size and severity.	[139]
	Animal	Treatment of *L. major*-infected NHPs (0.5 mg/kg CpG-ODN-D/A ID or IM once 3 days prior to infection, and once 3 days after infection).	Decrease in lesion size and severity.	[139]
	Animal	Treatment of *L. amazonensis*-infected NHPs (0.5 mg/kg CpG-ODN-D/A ID ID or IM once 3 days prior to infection, and once 3 days after infection).	Decrease in lesion size and severity.	[140]
	Animal	Treatment of SIV/*L. major*-infected NHPs (0.5 mg/kg CpG-ODN-D/A ID ID or IM once 3 days prior to infection, and once 3 days after infection).	Decrease in lesion size and parasite burden.	[140]
	Animal	Treatment of *L. major*-infected NHPs (single dose CpG-ODN-D35 1 mg/kg ID).	Decrease in lesion size and clinical score.	[141]
	Animal	Treatment of *L. major*-infected NHPs (single dose CpG-ODN-D35 0.5 mg/kg ID).	Increase in IFNγ-secreting PBMCs and decrease in lesion severity.	[142]
	Animal	Treatment of *L. major*-infected NHPs (single dose pro-CpG-ODN-D35 0.5 mg/kg ID).	Increase in IFNγ-secreting PBMCs and decrease in lesion severity.	[142]
Z-100 (TLR2 agonist) + MA	Animal	Treatment of *L. amazonensis*-infected Balb/c mice (Z-100 100 mg/kg/day IP × 2 weeks + MA 14 mg/kg/day IL × 2 weeks).	Limited therapeutic effect. Decrease in lesion size and severity.	[131]
Imiquimod + Sb	Animal	Treatment of *L. major*-infected Balb/c mice (imiquimod 5% ointment Top × 6 doses + SSG 100 mg/kg/day IP × 12 days).	Decrease in lesion size and enhancement of therapeutic effect of SSG.	[135]
	Human cohort (Ph3)	Treatment of 20 adult LCL patients in an area to which *L. peruviana* and *L. braziliensis* are endemic (imiquimod 5% ointment Top × 10 doses + MA 20 mg/kg/day IM × 20 days).	Increase in rapidity of treatment and decrease in residual scarring.	[143]
	Human cohort (Ph2)	Treatment of 12 adult LCL patients in an area to which *L. peruviana* and *L. braziliensis* are endemic (imiquimod 5% ointment Top × 10 doses + MA 20 mg/kg/day IM × 20 days).	Increase in 6-month cure rates.	[144]
	Human cohort (Ph3)	Treatment of 12 adult LCL patients in an area to which *L. peruviana* and *L. braziliensis* are endemic (imiquimod 5% ointment Top × 12 doses + MA 20 mg/kg/day IM × 14 days).	No clinical benefit of the addition of imiquimod to MA treatment.	[145]
	Human cohort (Ph2)	Treatment of 7 adult LCL patients (imiquimod 7.5% ointment Top × 10 doses + MA 20 mg/kg/day IV × 20 days).	Complete clinical cure and enhancement of MA therapeutic effect.	[146]
	Human cohort (Ph3)	Treatment of 39 adult LCL patients (imiquimod 5% ointment Top × 9 doses + MA 20 mg/kg/day IV × 20 days).	Decrease in disease duration and lesion area, though not statistically significant.	[147]
Imiquimod + PM	Animal	Treatment of *L. major*-infected Balb/c mice (imiquimod 5% /PM 15% ointment Top × 20 doses).	Enhancement of the therapeutic effect of PM.	[148]
Imiquimod + Itraconazole	Human cohort (Ph2)	Treatment of 14 adult LCL patients (imiquimod 5% ointment Top × 18 doses + itraconazole 200 mg/day PO × 6–8 weeks).	56% responsiveness rate to treatment.	[137]
Imiquimod + Dapsone	Human cohort (Ph2)	Treatment of 10 adult LCL patients (imiquimod 5% ointment Top × 18 doses + dapsone 2 mg/kg/day PO × 6–8 weeks).	70% responsiveness rate to treatment.	[137]
CpG-ODN-D (TLR9 agonist) + Sb	Animal	Treatment of *L. major*-infected NHPs (single dose CpG-ODN-D35 1 mg/kg ID + SSG 5 mg/kg/day IM × 5 days).	Decrease in lesion size and clinical score. Additive therapeutic effect with suboptimal SSG.	[141]

CL, cutaneous leishmaniasis; CpG-ODN, CpG oligodeoxynucleotide; ID, intradermal; IL, intralesional; IM, intramuscular; IP, intraperitoneal; IV, intravenous; LCL, localised cutaneous leishmaniasis; MA, meglumine antimoniate; NHP, non-human primate; PBMCs, peripheral blood mononuclear cells; PL, perilesional; PM, paromomycin; PO, per os/oral; Sb, pentavalent antimonials; SC, subcutaneous; SIV, simian immunodeficiency virus; SSG, sodium stibogluconate; Th1/Th2/Th17, T-helper 1/T-helper 2/T-helper 17; TLR, toll-like receptor; Top, topical. Combination immunotherapy and chemotherapy treatments are identified in grey.

**Table 5:** Animal and clinical studies assessing the use of modulators of cellular receptors and signalling for CL.

Therapy	Study Type	Study Design	Effect/Clinical Outcome	Reference
ITE (Aryl hydrocarbon receptor agonist)	Animal	Treatment of *L. major*-infected Balb/c mice (ITE single dose 30 nmol IL).	Reduction in lesion severity and parasitaemia and weakened Th2 response.	[157]
Glibenclamide/Glyburide (NLRP3 inhibitor)	Animal	Treatment of *L. mexicana*-infected Balb/c mice (Glibenclamide 80 mg/kg/day IP × 20 days).	Decreased lesion size and severity for drug-sensitive strains of parasites only.	[158]
	Animal	Treatment of *L. major*-infected Balb/c mice (Glibenclamide 80 mg/kg/day PO × 10 weeks).	Decrease in lesion size, TNF-α, IL-4, and IL-10.	[159]
	Animal	Treatment of *L. major/*LCMV co-infected C57BL/6 mice (5 μM Glyburide IP × 12–16 doses).	Decrease in lesion size and severity in co-infected mice, not reproduced in *Leishmania* mono-infected mice.	[160]
	Animal	Treatment of *L. braziliensis* infected RAG+CD8 mice (5 μM Glyburide IP × 12–16 doses).	Decrease in lesion size but not in parasitaemia.	[160]
MCC950 (NLRP3 inhibitor)	Animal	Treatment of *L. major/*LCMV co-infected C57BL/6 mice (5 mg/kg IP × 12–16 doses).	Decrease in lesion size and severity in co-infected mice, not reproduced in *Leishmania* mono-infected mice.	[160]
	Animal	Treatment of *L. braziliensis* infected RAG+CD8 mice (5 mg/kg IP × 12–16 doses).	Decrease in lesion size but not in parasitaemia.	[160]
Quercetin (NF-κB inhibitor)	Animal	Treatment of *L. major*-infected Balb/c mice (quercetin 14 mg/kg PO/IP/SC × 8 doses).	Increase in survival rate and similar therapeutic effect to MA.	[161]
	Animal	Treatment of *L. amazonensis*-infected Balb/c mice (quercetin 16 mg/kg/day PO × 30 days).	Decrease in lesion severity and parasite burden	[162]
	Animal	Treatment of *L. major*-infected Balb/c mice (quercetin 0.15% nanosomes ointment Top × 21 days).	Moderate decrease in lesion size.	[163]
	Animal	Treatment of *L. amazonensis*-infected Balb/c mice (quercetin nanosomes 0.4 mg/day PO × 51 days).	Decrease in lesion size and severity, and long-term disappearance of lesions and absence of parasites at sites of infection.	[164]
	Animal	Treatment of *L. braziliensis*-infected Syrian hamsters (quercetin 20 mg/kg/day PO × 5 weeks).	Decrease in lesion severity and parasite burden.	[165]
Chitin (CHI3L1 agonist)	Animal	Treatment of *L. major*-infected Balb/c mice (chitin 100 μg SC × 6 doses).	Decrease in lesion size and severity, and parasitic metastasis to visceral organs. Increase in TNF-α and IL-10.	[166]
Chitosan (CHI3L1 agonist)	Animal	Treatment of *L. major*-infected Balb/c mice (chitosan 100 μg SC × 6 doses).	Decrease in lesion size and severity, and parasitic metastasis to visceral organs.	[166]
	Animal	Treatment of *L. major*-infected Balb/c mice (chitosan 2–5 mg/mL hydrogel Top × 30 days).	No therapeutic effect.	[167]
	Animal	Treatment of *L. major*-infected Balb/c mice (chitosan platelets 100 μL IL × 6–7 doses).	Decrease in lesion size and severity and parasite load.	[168]
	Human cohort (Ph1)	Treatment of 10 adult LCL patients (chitosan nanocomposite film dressing changed 1x/week until clinical cure).	Significant or complete improvement of symptoms at 8 weeks post-treatment, and complete clinical cure of all patients by 12 weeks post-treatment. No recurrence at 6 months post-treatment.	[169]
HNP1	Animal	Treatment of *L. major*-infected Balb/c mice (HNP1 30 μg/week SC × 3 weeks).	Decrease in parasite load and induction of Th1 polarised response.	[170]
HNP1 pcDNA	Animal	Treatment of *L. major*-infected Balb/c mice (HNP1 pcDNA 100 μg/week SC × 3 weeks).	Decrease in parasite load and induction of Th1 response. More effective than folded HPN1.	[170]
HNP1 (*L. tarentolae)*	Animal	Treatment of *L. major*-infected Balb/c mice (2 × 10^5^/week *L. tarentolae* SC × 3 weeks).	Decrease in parasite load and induction of Th1 polarised response.	[171]
Glibenclamide (NLRP3 inhibitor) + Sb	Animal	Treatment of *L. mexicana*-infected Balb/c mice (Glibenclamide 80 mg/kg/day IP × 20 days, followed by Glibenclamide 60 mg/kg/day IP × 20 days + 75 mg/kg/day MA IP × 20 days).	Enhanced therapeutic effect of MA and decrease in lesion size.	[158]
Chitosan (CHI3L1 agonist) + AmB	Animal	Treatment of *L. major*-infected Balb/c mice (chitosan-AmB nanosomes 10 mg/kg IV × 5 doses).	No therapeutic effect.	[172]
	Animal	Treatment of *L. major*-infected Balb/c mice (chitosan-AmB nanosomes 10 mg/kg IP × 10 doses).	Decrease in lesion severity.	[173]
	Animal	Treatment of *L. amazonensis*-infected Balb/c mice (chitosan-AmB nanosomes 100 μL/day IV × 10 days).	Decrease in lesion severity and parasite burden, and metastasised parasites to visceral organs.	[174]
	Animal	Treatment of *L. major*-infected Balb/c mice (chitosan-AmB nanosomes 20 mg/kg IP × 10 doses).	Decrease in lesion severity and lymph node parasite load. More effective than MA.	[175]
	Animal	Treatment of *L. major*-infected Balb/c mice (chitosan-AmB nanosomes 100–200 μL/1–2 days IL/Top × 9–11 weeks).	Limited therapeutic effect. Decrease in parasite burden.	[168]
Chitosan (CHI3L1 agonist) + Betulinic acid	Animal	Treatment of *L. major*-infected Balb/c mice (chitosan-betulinic acid nanosomes 20 mg/kg IP × 20 doses).	Decrease in lesion severity and lymph node parasite load. More effective than MA.	[175]
	Animal	Treatment of *L. major*-infected Balb/c mice (chitosan-betulinic acid nanosomes 20 mg/kg IP × 20 doses).	Decrease in lesion severity and parasite burden.	[176]
Chitosan (CHI3L1 agonist) + Rifampicin	Animal	Treatment of *L. tropica*-infected Balb/c mice (chitosan-rifampicin nanosomes 12 mg/kg hydrogel Top × 21 days).	Decrease in lesion severity and parasite burden.	[177]
Chitosan (CHI3L1 agonist) + β-lapachone	Animal	Treatment of *L. major*-infected Balb/c mice (chitosan-β-lapachone nanosomes 20 mg/kg ointment Top × 21 days).	Decrease in lesion severity, but not in parasite load.	[178]
Chitosan (CHI3L1 agonist) + MSA (NO precursor)	Animal	Treatment of *L. amazonensis*-infected Balb/c mice (single dose chitosan-MSA 20 μL IL).	Decrease in lesion severity and absence of ulceration.	[179]

AmB, amphotericin B; CL, cutaneous leishmaniasis; IL, intralesional; IP, intraperitoneal; IV, intravenous; LCL, localised Cutaneous Leishmaniasis; LCMV, lymphocytic choriomeningitis virus; MA, meglumine antimoniate; MSA, mercaptosuccinic acid; PO, per os/oral; Sb, pentavalent antimonials; SC, subcutaneous; Top, topical. Combination immunotherapy and chemotherapy treatments are identified in grey.

**Table 6:** Animal and clinical studies assessing the use of enzyme inhibitors for CL.

Therapy	Study Type	Study Design	Effect/Clinical Outcome	Reference
Rapamycin (mTOR antagonist)	Animal	Treatment of *L. major*-infected Balb/c mice (rapamycin 1.5–10.2 μg/day IP × 10 days).	Decrease in lesion size and parasitaemia in the footpad and draining lymph node.	[184]
	Animal	Treatment of *L. tropica*-infected Balb/c mice (rapamycin 10.2 μg/day IP × 10 days).	Decrease in lesion size and parasitaemia.	[185]
GSK-2126458 (mTOR antagonist)	Animal	Treatment of *L. major*-infected Balb/c mice (GSK-2126458 1.5–10.2 μg/day IP × 10 days).	Decrease in lesion size and parasitaemia in the footpad and draining lymph node.	[184]
KU-0063794 (mTOR antagonist)	Animal	Treatment of *L. major*-infected Balb/c mice (KU-0063794 1.5–10.2 μg/day IP × 10 days).	No therapeutic effect.	[184]
AS-605240 (PI3K antagonist)	Animal	Treatment of *L. mexicana*-infected C57BL/6 mice (AS-605240 30 mg/kg/day IP × 2 weeks).	Decrease in lesion size and parasite load, comparable to SSG.	[186]
Harmine/ACB1801 (Multikinase inhibitor)	Animal	Treatment of resistant *L. major*-infected C57BL/6 mice (3 mg/kg/day IP × 16 days).	Reduced lesion development and parasite burden, and increased MHC II presentation signatures in the draining lymph nodes.	[187]
Tofacitinib (JAK 1/3 inhibitor)	Animal	Treatment of *L. braziliensis*-infected RAG^−/−^ Balb/c mice (tofacitinib 30 mg/kg/day IP or Top × 2 weeks).	Decrease in lesion severity, reduction in granzyme B expression by CD8^+^ T cells.	[188]
	Animal	Treatment of *L. major*-infected C57BL/6 mice (tofacitinib 30 mg/kg/day IP × 2 weeks).	No therapeutic effect.	[188]
Ibrutinib (Bruton tyrosine kinase inhibitor)	Animal	Treatment of *L. major*-infected Balb/c mice (ibrutinib 25 mg/kg/day PO × 16 days).	Decrease in lesion size and parasitaemia and increase in Th1 response.	[189]
Imatinib (Tyrosine kinase inhibitor)	Animal	Treatment of *L. amazonensis*-infected C57BL/6 mice (imatinib 200 mg/kg/day PO × 12–20 weeks).	Decrease in lesion size and severity.	[190]
	Animal	Treatment of *L. major*-infected Balb/c mice (10–150 mg/kg/day Top × 3 weeks).	Decrease in parasite load, particularly in the group receiving a 50 mg/kg dose.	[191]
Nor-NOHA (Arginase inhibitor)	Animal	Treatment of *L. major*-infected Balb/c mice (Nor-NOHA 1 mg/day IP × 2 weeks).	Decrease in parasite load, but non-significant effect on lesion size.	[192]
	Animal	Treatment of *L. amazonensis*-infected Balb/c mice (Nor-NOHA 10 μg IP × 16–24 doses).	Decrease in parasite load and lesion thickness, through a NO-dependent mechanism.	[193]
	Animal	Treatment of *L. major*-infected Balb/c mice (Nor-NOHA 10 μg/day IP × 50 days).	Decrease in footpad swelling and parasite burden.	[194]
L-arginine	Animal	Treatment of *L. major*-infected Balb/c mice (L-arginine 0.02 mg/day PO/Top/IV × 16 weeks).	Decrease in lesion size and severity in topical and injected groups. Decrease in liver and spleen parasite burden when administered orally.	[195]
	Animal	Treatment of *L. major*-infected Balb/c mice (L-arginine 10 mg IP × 6 doses).	Decrease in lesion severity and parasite burden. Restoration of T cell response.	[192]
Pentoxifylline (Phosphodiesterase inhibitor)	Animal	Treatment of *L. amazonensis*-infected Balb/c mice (pentoxifylline 8 mg/kg/12 h IP × 40–120 days).	Decrease in lesion severity and parasite burden.	[196]
Pravastatin (HMG-CoA inhibitor)	Animal	Treatment of *L. amazonensis*-infected Balb/c mice (pravastatin 20 mg/kg/day SC × 90 days).	Increase in complement-mediated phagocytosis and NO production and decrease in TNF production.	[197]
	Animal	Treatment of *L. amazonensis*-infected Balb/c mice, C57BL/6 mice, and Syrian hamsters (pravastatin 20 mg/kg/day SC × 30 days).	Increase in survival of BALB/c mice, of footpad thickness in both BALB/c and C57BL/6 mice, and decreased weight loss in C57BL/6 mice and Syrian hamsters.	[198]
Simvastatin (HMG-CoA inhibitor)	Animal	Treatment of *L. major*-infected Balb/c mice and C57BL/6 mice (simvastatin 20 mg/kg/day IP × 2 weeks).	Decrease in lesion size and ulceration in BALB/c, but not in C57BL/6. Decrease in parasite burden in all mice.	[199]
	Animal	Treatment of *L. major*-infected Balb/c mice and C57BL/6 mice (simvastatin 20 μL/day Top × 6–8 weeks).	Decrease in lesion size and ulceration, and parasite load.	[199]
Rapamycin (mTOR antagonist) + AmB	Animal	Treatment of *L. tropica*-infected Balb/c mice (rapamycin 10.2 μg/day IP × 10 days + AmB 8 mg/kg/day IP × 10 days).	No therapeutic effect.	[185]
Rapamycin (mTOR antagonist) + MA	Animal	Treatment of *L. tropica*-infected Balb/c mice (rapamycin 10.2 μg/day IP × 10 days + MA 200 mg/kg/day SC × 10 days).	No therapeutic effect.	[185]
AS-605240 (PI3K antagonist) + Sb	Animal	Treatment of *L. mexicana*-infected C57BL/6 mice (AS-605240 30 mg/kg/day IP × 2 weeks + SSG 20 mg/kg/day).	Enhancement of therapeutic effect of suboptimal SSG.	[186]
Pentoxifylline (Phosphodiesterase inhibitor) + MTF	Human cohort (Ph2)	Treatment of 22 adult LCL/MCL patients from a region to which *L. braziliensis* is endemic (pentoxifylline 1200 mg/day PO + MTF 100 mg/day PO × 20 days for LCL or 28 days for MCL).	Increased cure rate for CL patients and decreased risk of adverse effects.	[200]
Pentoxifylline (Phosphodiesterase inhibitor) + Sb	Human cohort (Ph2)	Treatment of 21 adult LCL/MCL patients from a region to which *L. braziliensis* is endemic (pentoxifylline 1200 mg/day PO + MA 20 mg/kg/day IV × 20 days for LCL or 30 days for MCL).	Increased cure rate for CL patients.	[200]
	Human cohort (Ph2)	Treatment of 36 adult LCL patients from a region to which *L. panamensis* is endemic (pentoxifylline 1200 mg/day PO + MA 20 mg/kg/day IV × 30 days).	No additional therapeutic effect.	[201]
	Human cohort (Ph2)	Treatment of 12 adult MCL patients from a region to which *L. braziliensis* is endemic (pentoxifylline 1200 mg/day PO + MA 20 mg/kg/day IV × 30 days).	Decrease in average healing time and relapse at 2 years post-treatment. Enhanced efficacy of a single course of MA.	[202]
	Human cohort (Ph2)	Treatment of 82 adult LCL patients from a region to which *L. braziliensis* is endemic (pentoxifylline 1200 mg/day PO + MA 20 mg/kg/day IV × 30 days).	No additional therapeutic effect.	[203]
	Human cohort (Ph2)	Treatment of 10 adult MCL patients from a region to which *L. braziliensis* is endemic (pentoxifylline 1200 mg/day PO + MA 20 mg/kg/day IV × 30 days).	Complete clinical cure of almost all patients.	[204]

CL, cutaneous leishmaniasis; IP, intraperitoneal; IV, intravenous; LCL, localised cutaneous leishmaniasis; MA, meglumine antimoniate; MCL, mucocutaneous leishmaniasis; MTF, miltefosine; PO, per os/oral; Sb, pentavalent antimonials; SC, subcutaneous; SSG, sodium stibogluconate; Top, topical. Combination immunotherapy and chemotherapy treatments are identified in grey.

**Table 7:** Animal and clinical studies assessing the use of anti-inflammatory and antioxidant agents for CL.

Therapy	Study Type	Study Design	Effect/Clinical Outcome	Reference
Acetylsalicylic acid	Animal	Treatment of *L. major*-infected Balb/c mice (acetylsalicylic acid 400 mg/kg/day PO × 13 weeks).	Decrease in lesion severity and parasitic metastasis to visceral organs and increase in NO production.	[210]
Gentisic acid	Animal	Treatment of *L. amazonensis*-infected Balb/c mice (gentisic acid 30 mg/kg IL × 5 doses).	Decrease in lesion size and parasite burden.	[211]
P-coumaric acid	Animal	Treatment of *L. amazonensis*-infected Balb/c mice (P-coumaric acid 30 mg/kg IL × 5 doses).	Decrease in lesion severity, but not in parasite load.	[211]
Gallic acid	Animal	Treatment of *L. major*-infected Balb/c mice (gallic acid 3% ointment Top × 21 days).	Decrease in lesion severity and parasite burden.	[212]
Ellagic acid	Animal	Treatment of *L. major*-infected Balb/c mice (ellagic acid 3% ointment Top × 21 days).	Decrease in lesion severity and parasite burden.	[212]
Ursolic acid	Animal	Treatment of *L. amazonensis*-infected Syrian hamsters (ursolic acid 0.2–0.5% ointment Top × 4 weeks).	Decrease in lesion size and severity.	[213]
Berberine chloride	Animal	Treatment of *L. major*-infected C57BL/6 mice (berberine chloride 7.5 mg/kg ointment Top × 35 days).	Decrease in lesion size and parasite load and decreased inflammatory cell infiltration.	[214]
Solamargine + Solasonine	Animal	Treatment of *L. mexicana*-infected Balb/c mice (solamargine 45.1%/Solasonine 44.4% ointment Top × 21 days).	Moderate decrease in lesion size.	[215]
Salicylic acid + Potassium nitrite	Animal	Treatment of *L. major*-infected Balb/c mice (salicylic acid 2%/potassium nitrite 2–10% ointment Top × 10 days).	No therapeutic effect.	[216]
	Human cohort (Ph2)	Treatment of 10 LCL patients caused by *L. tropica* (salicylic acid 2%/potassium nitrite 2–5% ointment Top × 4 weeks).	No/limited therapeutic effect.	[216]
Ascorbic acid + Potassium nitrite	Animal	Treatment of *L. major*-infected Balb/c mice (ascorbic acid 2%/potassium nitrite 2–10% ointment Top × 10 days).	No therapeutic effect.	[216]
	Human cohort (Ph2)	Treatment of 26 LCL patients caused by *L. tropica* (ascorbic acid 2%/potassium nitrite 2–5% ointment Top × 4 weeks).	No/limited therapeutic effect.	[216]
Potassium chloride + Potassium nitrite	Animal	Treatment of *L. major*-infected Balb/c mice (potassium chloride 2%/potassium nitrite 2–10% ointment Top × 10 days).	No therapeutic effect.	[216]
	Human cohort (Ph2)	Treatment of 4 LCL patients caused by *L. tropica* (potassium chloride 2%/potassium nitrite 2–5% ointment Top × 4 weeks).	No/limited therapeutic effect.	[216]
Gallic acid + AmB	Animal	Treatment of *L. major*-infected Balb/c mice (gallic acid 3%/AmB 1.5% ointment Top × 21 days).	Decrease in lesion severity and parasite burden, and slight enhancement of AmB therapeutic effect.	[212]
Ellagic acid + AmB	Animal	Treatment of *L. major*-infected Balb/c mice (ellagic acid 3%/AmB 1.5% ointment Top × 21 days).	Decrease in lesion severity and parasite burden, and slight enhancement of AmB therapeutic effect.	[212]

AmB, amphotericin B; CL, cutaneous leishmaniasis; IL, intralesional; LCL, localised cutaneous leishmaniasis; PO, per os/oral; Top; topical. Combination immunotherapy and chemotherapy treatments are identified in grey.

**Table 8:** Animal studies assessing the use of cell therapy for CL.

Therapy	Study Type	Study Design	Effect/Clinical Outcome	Reference
MSCs	Animal	Treatment of *L. amazonensis*-infected Balb/c mice (1 × 10^5^ MSCs IL/IV × 2 doses).	No difference in lesion progression, increase in parasite load.	[218]
	Animal	Treatment of *L. major*-infected Balb/c mice (single dose of 1 × 10^6^ MSCs IL).	Decrease in lesion severity but not in parasite burden.	[219]
BM-DCs + IL-12	Animal	Treatment of *L. major*-infected C3HeB mice (1 × 10^6^ BM-DCs SC × 6 doses + 0.2 μg IL-12 SC × 6 doses).	Limited therapeutic effect; increase in Th1 cytokines but no promotion of healing.	[220]
MSCs + Sb	Animal	Treatment of *L. amazonensis*-infected Balb/c mice (single dose of 1 × 10^6^ MSCs IL + MA 30 mg/kg/day IM × 3 weeks).	Decrease in lesion severity and parasite burden.	[219]

BM-DCs, bone marrow dendritic cells; IL, intralesional; IM, intramuscular; IV, intravenous; MA, meglumine antimoniate; MSCs, bone marrow mesenchymal stromal cells; Sb, pentavalent antimonials. Combination immunotherapy and chemotherapy treatments are identified in grey.

**Table 9:** Animal and clinical studies assessing the use of therapeutic vaccination for CL.

Type	Immunogen	Study Type	Study Design	Effect/Clinical Outcome	Reference
Inactivated	*L. amazonensis*	Human cohort (Ph3)	Treatment of 53 LCL patients (vaccine 36–172 μg/day IM × 10 days).	No clinical benefit.	[221]
	Leishvacin©	Human cohort (Ph2/3)	Treatment of 19 LCL patients (vaccine 500 μg/week IM × 12 weeks).	Minimal decrease in remission time in comparison to Sb treatment.	[222]
	*L. amazonensis* + BCG	Human cohort (Ph2)	Treatment of 124 adolescent/adult LCL patients (6.4 × 10^8^ promastigotes + 0.01–0.02 mg BCG ID × 3 doses).	Cure rate similar to Sb treatment and increase in Th1 response observed in PBMCs.	[223]
		Clinical case	Treatment of a 40-year-old male patient with treatment-resistant LCL caused by *L. amazonensis* (6.4 × 10^8^ promastigotes + 0.075 mg BCG ID × 3 doses).	Full clinical recovery and stimulation of Th1 response.	[224]
	*L. braziliensis* + BCG	Human cohort (Ph1/2)	Treatment of 4 MCL and 3 ADCL patients (6.4 × 10^8^ promastigotes + 0.01 mg BCG ID × 10 doses).	Complete remission of MCL after 5-9 doses, and of DCL from 7–10 doses.	[225]
	*L. mexicana* + BCG	Human cohort (Ph2)	Treatment of >5000 LCL patients (6.4 × 10^8^ promastigotes + 0.01–0.02 mg BCG ID × 3 doses).	95.7% cure rate with use of immunotherapy.	[226]
Live attenuated	BCG	Human cohort (Ph4)	Treatment of 12 adult LCL/MCL patients (0.01–0.02 mg BCG ID × 3–4 doses).	38% cure rates of patients with CL.	[223]
Whole lysate	SLA + Pam3Cys + R848	Animal	Treatment of *L. major*-infected Balb/c mice (SLA 25 μg + Pam3Cys 10 μg + R848 10 μg SC × 3 doses).	Decrease in lesion severity and parasite burden; increase in IFN-γ and NO production.	[227]
	SLA + ONO-4007	Animal	Treatment of *L. amazonensis*-infected Balb/c mice (250 μg SLA + 50 mg/kg ONO-4007 SC × 7 doses).	Decrease in lesion size and severity, and increase in IFN-γ.	[133]
Subunit (Protein)	TSA, LmSTI1, LeIF and HSP83 + GM-CSF	Clinical case	Treatment of a 45-year-old male patient with treatment-resistant LCL (antigens 5–10 mg/month + GM-CSF 50 μg/month SC × 3 months).	Resolution of all lesions without relapse at 2 years post-treatment.	[228]
		Human cohort (Ph2)	Treatment of 6 treatment-refractory MCL patients (antigens 5 mg/month + GM-CSF 50 μg/month SC × 3 months).	Clinical remission of 5/6 patients without relapse at 5 years post-treatment.	[229]
	L110f + MPLA	Animal	Treatment of *L. major*-infected Balb/c mice (L110f 5 μg/week SC + MPL 20 μg/week SC × 6 weeks).	No therapeutic effect.	[230]
	L110f + MPLA + CpG-ODN-B	Animal	Treatment of *L. major*-infected Balb/c mice (L110f 5 μg/week SC + MPL 20 μg/week SC + CpG-ODN-K 50 μg/week SC × 6 weeks).	Decrease in lesion size and parasite burden and increase in protective T cell response.	[230]
	L110f + GLA	Animal	Treatment of *L. major*-infected Balb/c mice (L110f 5 μg/week SC + GLA 20 μg/week SC × 6 weeks).	Limited therapeutic effect.	[230]
	L110f + GLA + CpG-ODN-B	Animal	Treatment of *L. major*-infected Balb/c mice (L110f 5 μg/week SC + GLA 20 μg/week SC + CpG-ODN-B 50 μg/week SC × 6 weeks).	Decrease in lesion size and parasite burden.	[230]
	L110f + CpG-ODN-B	Animal	Treatment of *L. major*-infected Balb/c mice (L110f 5 μg/week SC + CpG-ODN-B 50 μg/week SC × 6 weeks)	Limited therapeutic effect and decrease in CD4^+^ T cell response.	[230]
	NH36 + Saponin	Animal	Treatment of *L. amazonensis*-infected Balb/c mice (NH36 100 μg/week + saponin 100 μg/week SC × 3 weeks).	Decrease in lesion severity and parasite burden.	[231]
Subunit (DNA)	GP46 DNA	Animal	Treatment of *L. major*-infected Balb/c mice (single dose GP46 DNA 50–100 μg IM).	Decrease in lesion severity and increase in protective Th1 cytokines.	[232]
Viral vector	ChAd63-KH (KMP-11 and HASPB)	Human cohort (Ph2a)	Treatment of 23 Sudanese PKDL patients (single dose 1–7.5 × 10^10^ viral particles IM).	50% of patients showed clinical improvement.	[233]
Inactivated	*L. amazonensis* + Sb	Human cohort (Ph3)	Treatment of 38 LCL patients (vaccine 36–172 μg/day IM × 10 days + MA 1 mg/kg/day IM × 10 days).	Decrease in average healing time and required dose of Sb.	[221]
		Human cohort (Ph1)	Treatment of 47 LCL patients (vaccine 0.5 mL/day SC × 10 days + MA 8.5 mg/kg/day IM × 10 days).	100% cure rate in combination therapy group, as compared to only 8.2% in the control group.	[234]
	Leishvacin© + Sb	Human cohort (Ph2)	Treatment of 54 LCL patients (vaccine 100 μL–500 μg/day SC × 5 days with 10-day intervals until clinical cure + MA 1 mL/5kg/day).	No decrease in healing time. Increase in Th1/Th2 response in PBMCs.	[235]
	*L. amazonensis* + *L. braziliensis* + BCG + Sb + AmB	Clinical case	Treatment of a 22-year-old male patient with treatment-resistant DL (*L. amazonensis* 600 μg + *L. braziliensis* 600 μg + BCG 400 μg SC + standard chemotherapy).	Temporary remission of lesions was attained along with an increase in circulatory PBMCs and NK cells.	[236]
	Alum/ALM + BCG + Sb	Human cohort (Ph2)	Treatment of 15 patients with persistent PKDL (alum/ALM 100 μg SC 4×/week + BCG 0.01 mL IM 4×/week + SSG 20 mg/kg/day IM/IV × 40 days).	Complete clinical cure and no relapse 90 days post-treatment.	[237]
Live attenuated	BCG + Sb	Human cohort (Ph3)	Treatment of 47 LCL patients (BCG 100 μg/day ID + MA 1 mL/5kg/day IM × 10 days).	Decrease in mean cure time comparatively to Sb alone.	[221]
Subunit (Protein)	Leish-F1 (TSA, LmSTI1, LeIF) + MPL + Sb	Human cohort (Ph1)	Treatment of 27 LCL patients (Leish-F1 5–20 μg + MPL 25 μg SC × 3 doses + MA 10 mg/kg/day × 10 days with 11 day intervals until cure).	80% cure rates in CL patients comparatively to 38% and 50% in adjuvant and placebo controls.	[238]

AmB, amphotericin B; BCG, Bacillus Calmette-Guérin; CpG-ODN, CpG oligodeoxynucleotides; DL, disseminated leishmaniasis; GM-CSF, granulocyte-macrophage colony-stimulating factor; GLA, glucopyranosyl lipid adjuvant; ID, intradermal; IM, intramuscular; LCL, localised cutaneous leishmaniasis; MA, meglumine antimoniate; MCL, mucocutaneous leishmaniasis; MPLA, monophosphoryl lipid A; NK, natural killer cells; PBMCs, peripheral blood mononuclear cells; Sb, pentavalent antimonials; SC, subcutaneous; SLA, soluble *Leishmania* antigen; SSG, sodium stibogluconate; Th1/Th2, T-helper 1/T-helper 2. Combination immunotherapy and chemotherapy treatments are identified in grey.
